# Clinical Effects of L-Carnitine Supplementation on Physical Performance in Healthy Subjects, the Key to Success in Rehabilitation: A Systematic Review and Meta-Analysis from the Rehabilitation Point of View

**DOI:** 10.3390/jfmk6040093

**Published:** 2021-11-04

**Authors:** Michele Vecchio, Rita Chiaramonte, Gianluca Testa, Vito Pavone

**Affiliations:** 1 Department of Biomedical and Biotechnological Sciences, Section of Pharmacology, University of Catania, 95123 Catania, Italy; 2 Rehabilitation Unit, “AOU Policlinico Vittorio Emanuele”, 95123 Catania, Italy; 3 Department of General Surgery and Medical Surgical Specialties, Section of Orthopaedics and Traumatology, University Hospital Policlinico “Rodolico-San Marco”, University of Catania, 95123 Catania, Italy; gianluca.testa@unict.it (G.T.); vpavone@unict.it (V.P.)

**Keywords:** carnitine, rehabilitation, physical activity

## Abstract

L-carnitine supplementation improves body strength, sports endurance and exercise capacity, as well as delaying the onset of fatigue. The aim of this study was to identify the correct dosage of supplementation to obtain improvements in physical performance and evaluate the changes related to L-carnitine supplementation in specific metabolic parameters, such as serum lactate, VO_2_, serum total and free carnitine at rest and after physical activities, in healthy subjects. The search was conducted on PubMed, EMBASE, Cochrane Library, Scopus and Web of Science and identified 6404 articles with the keywords: “carnitine” AND “exercises” OR “rehabilitation” OR “physical functional performance” OR “physical activity” OR “sports” OR “health” OR “healthy”. A total of 30 publications met the inclusion criteria and were included in the systematic review. The meta-analysis did not show any significant differences in serum lactate values at rest and after exercise in healthy subjects who took L-carnitine supplementation (*p* > 0.05). On the contrary, L-carnitine administration significantly changed maximal oxygen consumption (VO_2_) at rest (*p* < 0.005), serum free and total carnitine at rest and after exercise (*p* < 0.001). The dosage of supplementation that obtained a significant change in serum total carnitine was 2 g/dL for 4 weeks at rest, 1 g/dL for 3 weeks after exercise, and in serum free carnitine was 2 g/dL for 3 weeks and 2 g/dL for 4 weeks at rest. Based on our study, serum total and free carnitine at rest and after exercise, and VO_2_ at rest could be used to clinically follow individuals during physical activity and rehabilitation programs. Moreover, the supplementation should have a correct dosage to have maximum effect. Other robust trials are needed to find the best dosage to obtain positive results in metabolic parameters and in physical performance.

## 1. Introduction

L-carnitine (L 3-hydroxy-4-N-trimethyl amino-butyric acid) plays an essential role in enhancing endurance and recovery from fatigue [1].

The beneficial effects of both acute and chronic L-carnitine supplementation in several pathologic conditions are well described, such as infectious diseases (such as the Human immunodeficiency virus HIV), hemodialysis, cancer cachexia and dystonia [2,3,4,5,6]. Its intake has beneficial effects on healthy subjects, too. In the elderly, it reduces fatigue sensation and improves physical, mental and cognitive function as well as reducing fatigue [7]. In athletes, it facilitates the recovery process and increases blood flow and oxygen supply to muscle tissue [8]. In addition, it alleviates muscle injury and reduces markers of cellular damage, decreasing free radical formation [8].

The aim of the meta-analysis was to identify the correct dosage of supplementation to obtain improvements in physical performance and to investigate the changes related to L-carnitine supplementation in specific metabolic parameters, such as blood lactate, VO_2_, serum total and free carnitine at rest and after physical activity in healthy subjects.

## 2. Methods

### 2.1. Search Strategy

The search was performed on the following medical electronic databases: PubMed, EMBASE, Cochrane Library, Scopus and Web of Science. The reference list of the related articles was also used to search for other eligible papers. The search strategy was conducted from December 2019 to January 2021 with the following terms and keywords: “carnitine” AND “exercises” OR “rehabilitation” OR “physical functional performance” OR “exercises” OR “sport” OR “health” OR “healthy”.

**Figure 1:**
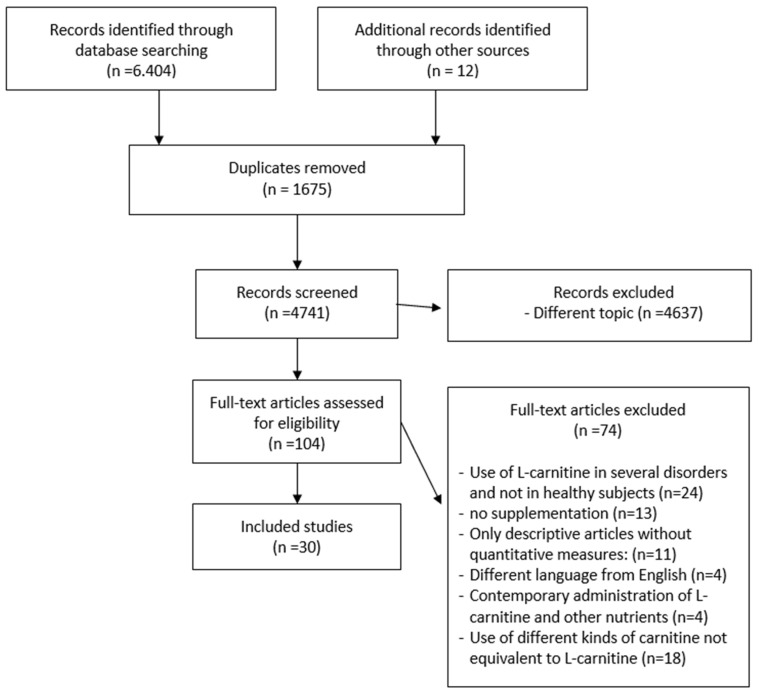
Flowchart of the process of initial literature search and extraction of studies meeting the inclusion criteria.

The search identified 6404 articles from 1976 to 2020. The reviewers analyzed 104 full texts. The eligibility for study inclusion was assessed independently. There were 30 publications that met the criteria and were included in the study (Table 1). The systematic review included 15 articles used for the meta-analysis. Data extraction included authors, year, sample characteristics, comparison and control groups, physical performance or rehabilitation outcomes, periodic assessments and follow-up, and outcomes identified after supplementation. After the removal of duplicates (*n* = 1675), several articles (*n* = 4637) were excluded because they were irrelevant on the basis of title and abstract or were irrelevant to the research question. A further 74 articles were excluded for other reasons: use of L-carnitine in several disorders and not in healthy subjects (*n* = 24), no supplementation of L-carnitine (endogenous carnitine) (*n* = 13), descriptive articles about the use of supplementation without quantitative measures (no concentration, dosage and duration of administration) (*n* = 11), not written in English (*n* = 4), contemporary administration of L-carnitine and other nutrients (*n* = 4), use of different kinds of L-carnitine not equivalent to L-carnitine (*n* = 18)(Figure 1).

### 2.2. Study Selection

Studies were included in the review if they respected the following criteria: (a) design: randomized controlled trial (RCT), prospective and retrospective studies, only published data were permitted; (b) language: original article in English; (c) participants: healthy adults, including the elderly and athletes, who used dietary supplementation with L-carnitine; (d) intervention: rehabilitative program and physical exercise; (e) comparison: no nutrient supplementation, or different dosage, or assessments at rest or after training; (f) outcomes: improvement of physical performance and changes in metabolic parameters.

Animal studies and papers without L-carnitine supplementation or papers with children or no healthy people as participants in the studies with L-carnitine supplementation were excluded. Any duplicates were also excluded.

The meta-analysis considered the studies with numeric values and quantitative data useful to quantify the outcomes and the effects of L-carnitine in healthy adults. These values were the maximal oxygen uptake (VO_2_) and various blood parameters at rest and after exercise, such as plasma lactate, serum total and free carnitine.

This review is registered on PROSPERO: CRD42021229692.

### 2.3. Data Collection Process, Data Extraction and Outcomes

The titles and abstracts of studies retrieved using the search strategy and those from additional sources were screened independently by two authors to identify studies that potentially met the inclusion criteria outlined above.

Selected full texts were then reviewed and included in the systematic review and in the meta-analysis, following the Preferred Reporting Items for Systemic Reviews and Meta-analyses (PRISMA) statement [9], the Meta-analyses of Observational Studies (MOOSE) checklist [10] and the PICOS (population, intervention, comparison, outcome and study design) criteria [11] shown in Table 1: Participants were adults; Intervention was based on the supplementation of L-carnitine; Comparator was any comparator; Outcomes included clinical assessments and blood tests; and Study design included RCTs, and retrospective and prospective studies. Any disagreement over the eligibility of particular studies was resolved through discussion between the authors.

The main outcome was to show the effects of dietary L-carnitine intake in healthy subjects, adults, the elderly and athletes that could be useful to improve physical performance and obtain better results in rehabilitation. The secondary outcome was to define the metabolic changes induced by L-carnitine addition and the best dosage according to the current literature.

### 2.4. Risk of Bias

Two authors independently assessed the risk of bias of the included studies using the Cochrane risk of bias tool [12]. The assessments of risk of bias included random sequence generation, allocation concealment, blinding of participants, blinding of outcome assessment, incomplete outcome data, selective reporting and other biases. The adequacy of included studies as low, unclear, moderate or high risk for each study was analyzed by the authors (Table 2).

### 2.5. Quality of Outcomes

The Grading of Recommendations Assessment, Development and Evaluation (GRADE) guidelines [13,14,15,16,17] for systematic reviews were used to evaluate the quality of the results. The rating of the quality of the study outcome was carried out to indicate the degree of certainty (high, moderate, low or very low) of the total effect estimates (Table 3).

### 2.6. Meta-Analysis Calculations

The Statistical Package for Social Sciences (SPSS, Version 18.0 for Windows; SPSS Inc., Chicago, IL, USA) was used for data analysis.

A synthesis of the findings from the included studies met the PICOS criteria. Summaries of intervention effects for each study were calculated with the standardized mean differences for continuous outcomes.

For the studies with the same type of intervention and comparator and with the same outcome measure, a random-effects meta-analysis with standardized mean differences for continuous outcomes was used, and 95% confidence intervals and two-sided *p* values for each outcome were calculated.

Heterogeneity was assessed using the inconsistency test (I^2^). The I^2^ verified the impact of study heterogeneity on the results of the meta-analysis; an I^2^ value greater than 50% was indicative of substantial heterogeneity. An I^2^ value < 25% was indicative of a low risk of heterogeneity, a value between 25% and 50% was indicative of a moderate level of heterogeneity and >50% was considered statistically significant between the included studies [18].

The sensitivity analysis was based on study quality. The stratified meta-analyses explored the heterogeneity according to: study quality; study populations; the logistics of intervention provision; and intervention content. The random-effect model estimated the combined effect sizes [19]. The quality of identified studies followed the methods of the Cochrane Collaboration [20], and publication bias was examined using funnel plots.

## 3. Results

### 3.1. Variations of Experimental Conditions across the Studies

The meta-analysis assessed the modifications in metabolic parameters after different dosages of L-carnitine and the differences at rest and after exercise.

The effectiveness of L-carnitine intake on levels of serum lactate is showed in Table 4 and Table 5, VO_2_ in Table 6 and serum total and free carnitine in Table 7 and Table 8.

All study groups were not homogeneous for relevant general features, such as age, sex and physical performance. Other characteristics of the studies were the supplementation dosage and duration of dietary intake, metabolic parameters used for the assessment of physical performance after L-carnitine intake and follow up (Table 1). In addition, the participants were submitted to different kinds and durations of physical exercise. Only a few studies were included in the meta-analysis because of sample inhomogeneity and the lack of quantitative measures.

### 3.2. Participants, Interventions and Comparators

The studies included in the systematic review met the PICOS criteria [11]. This systematic review included original studies on L-carnitine supplementation in healthy subjects (Table 1). In all of the studies, the participants were healthy adults, the elderly or athletes. The studies of obese subjects with metabolic disorders were excluded, but healthy overweight individuals were included.

The studies described the effects of dietary L-carnitine supplementation, comparing no intake, different dosages, different periods of follow-up, at rest and after physical exercise.

The outcomes included clinical assessments and metabolic parameters. All the studies used validated measurement tools and clearly showed their results. The design of the studies was RCT, retrospective and prospective studies, according to the recommendations of the Oxford Centre for Evidence-Based Medicine (Table 1). The meta-analysis focused on the quantitative results of metabolic parameters that were reported in more than one article.

### 3.3. Meta-Analysis Results

A total of 15 studies were included in the meta-analysis (Table 4, Table 5, Table 6, Table 7 and Table 8). Pooling of data within the meta-analysis revealed that several measures including serum lactate at rest (Table 4) and after exercise (Table 5) did not present significant variations with and without L-carnitine administration (*p* > 0.05). On the contrary, significant variations of VO_2_ at rest (*p* < 0.005) (Table 6), and serum total and free carnitine at rest and after exercise (Table 7 and Table 8) (*p* < 0.001) and after L-carnitine administration were found.

The dosage of supplementation that obtained a significant change in serum total carnitine was 2 g/dL for 4 weeks at rest and 1 g/dL for 3 weeks after exercise (Table 7), and in serum free carnitine it was 2 g/dL for 3 weeks and 2 g/dL for 4 weeks at rest (Table 8). The data were not sufficient to obtain statistically significant values for VO_2_ and plasma lactate.

### 3.4. Heterogeneity and Publication Bias

**Figure 2:**
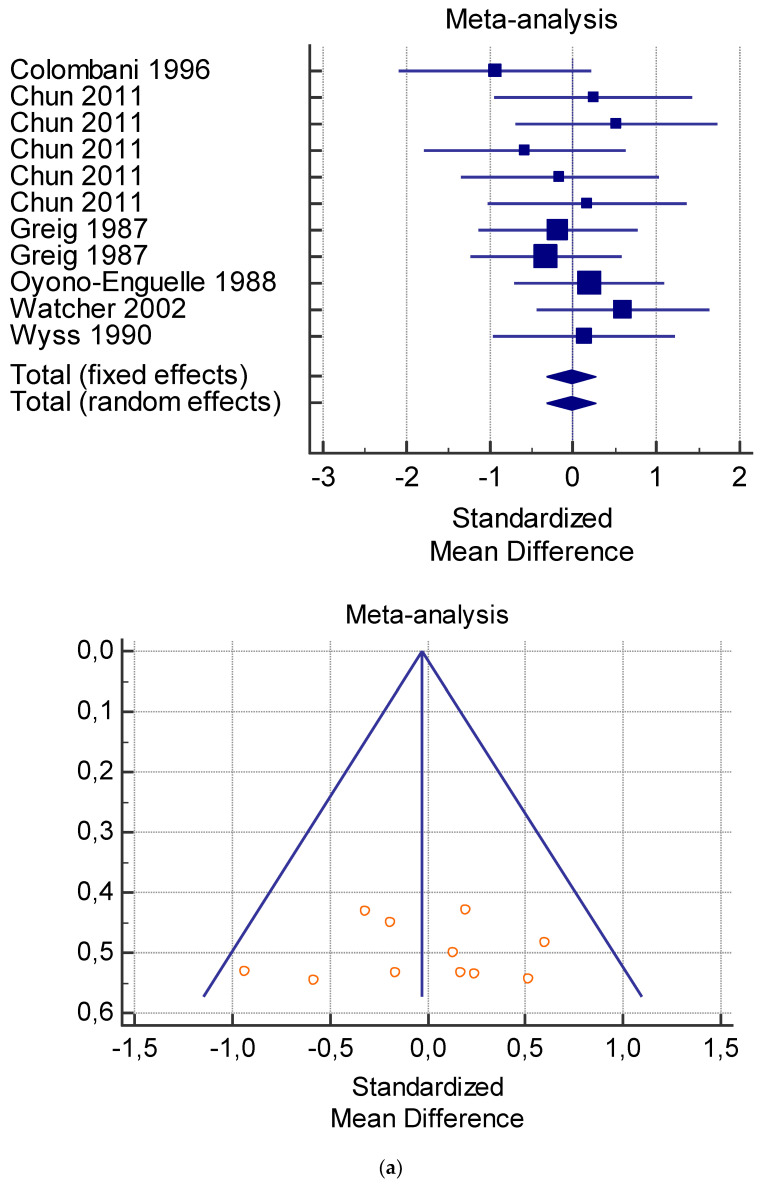

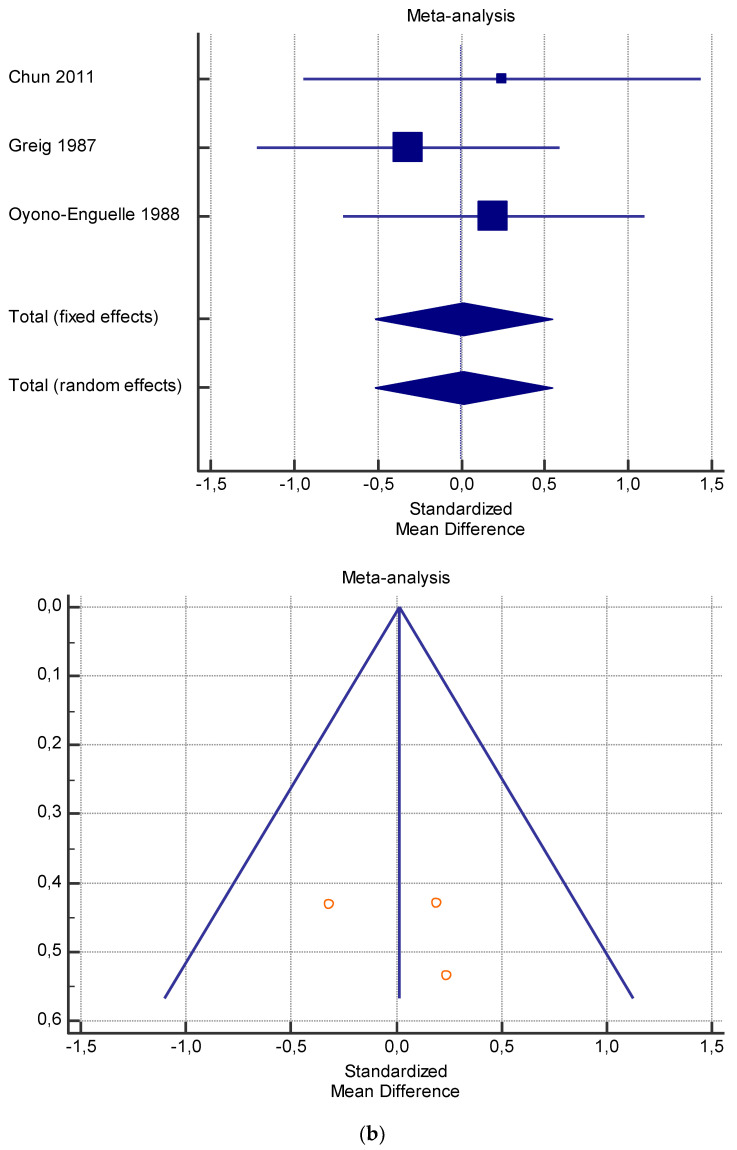
(**a**) Graphic correlation to Table 4: Plasma lactate (mmol/L) at rest with and without L-carnitine supplementation. (**b**) Graphic correlation to Table 4: Plasma lactate (mmol/L) after 2 g/dL for 4 weeks of L-carnitine supplementation.

**Figure 3:**
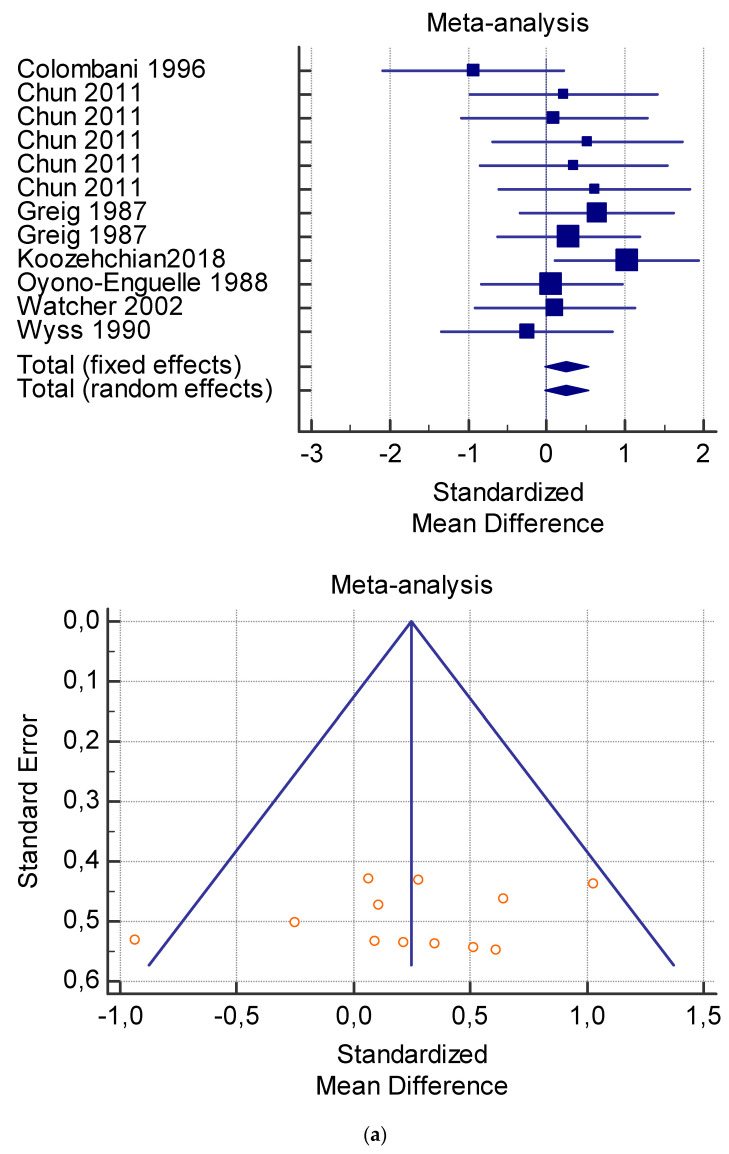

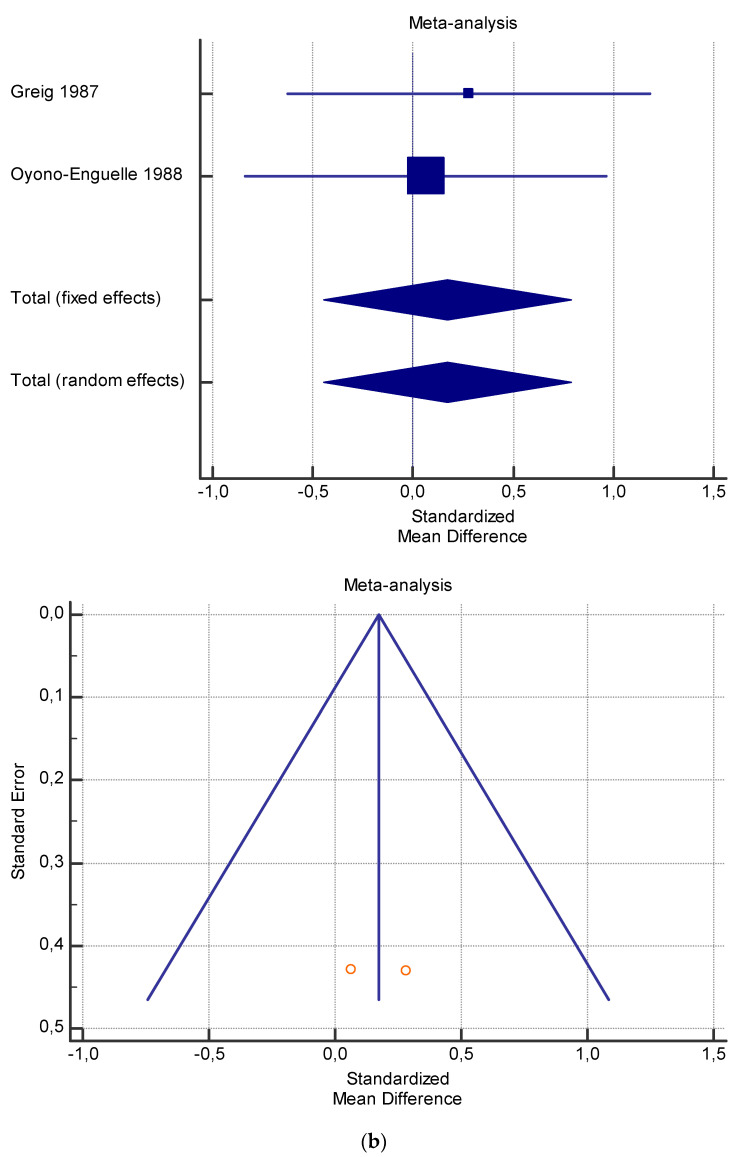
(**a**) Graphic correlation to Table 5: Plasma lactate (mmol/L) after exercise with and without and L-carnitine supplementation. (**b**) Graphic correlation to Table 5: Plasma lactate (mmol/L) after exercise after 2 g/dL for 4 weeks of L-carnitine supplementation.

**Figure 4:**
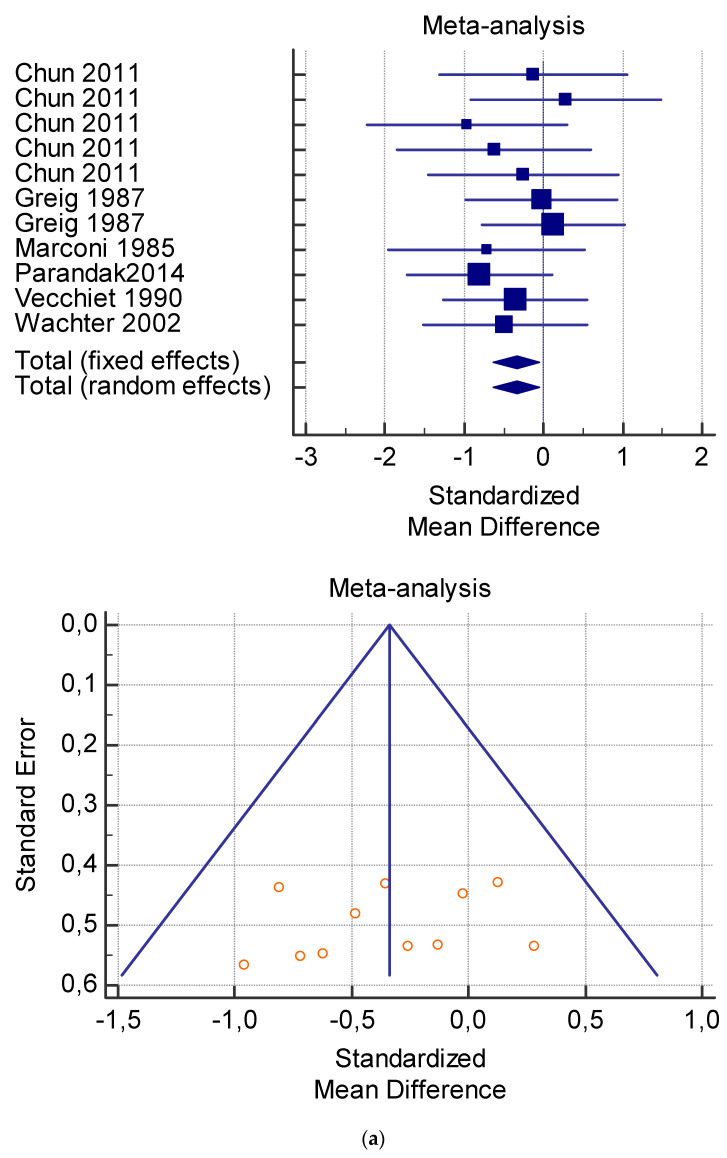

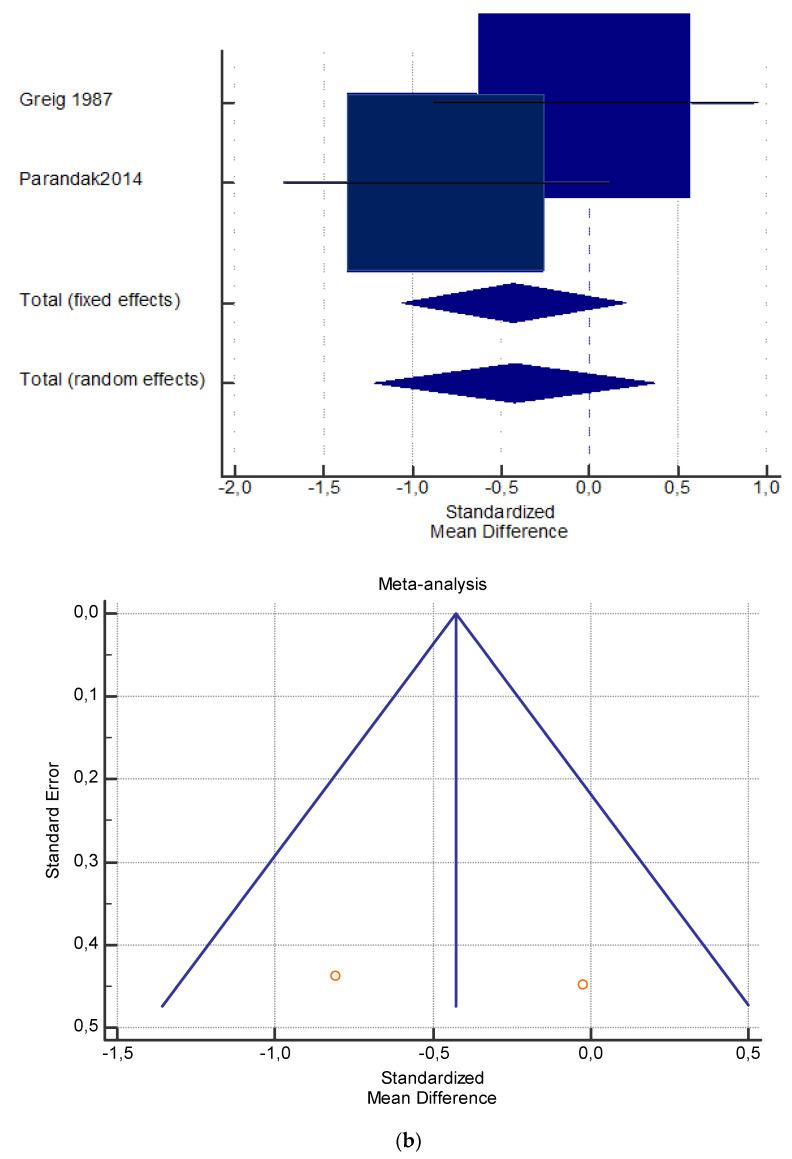

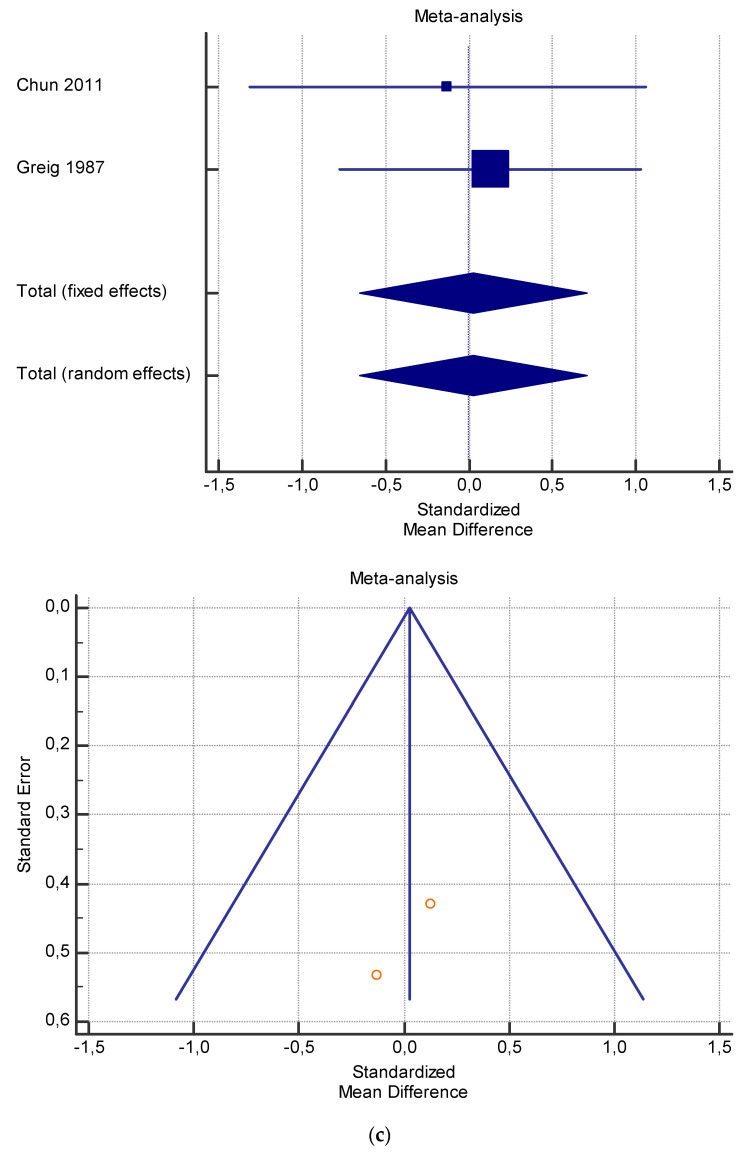
(**a**) Graphic correlation to Table 6: Maximal oxygen consumption VO_2_ (mL/min/kg) at rest with and without the L-carnitine supplementation. (**b**) Graphic correlation to Table 6: Maximal oxygen consumption VO_2_ (mL/min/kg) at rest after 2 g/dL for 2 weeks of L-carnitine supplementation. (**c**) Graphic correlation to Table 5: Maximal oxygen consumption VO_2_ (mL/min/kg) at rest after 2 g/dL for 4 weeks of L-carnitine supplementation.

**Figure 5:**
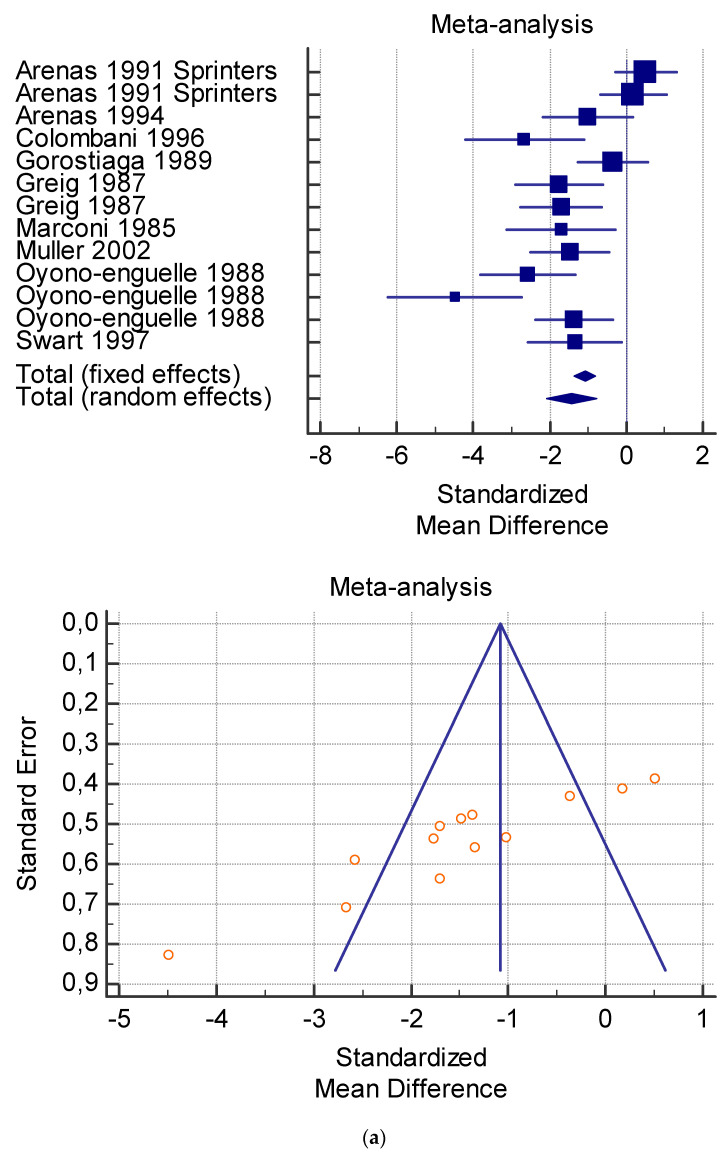

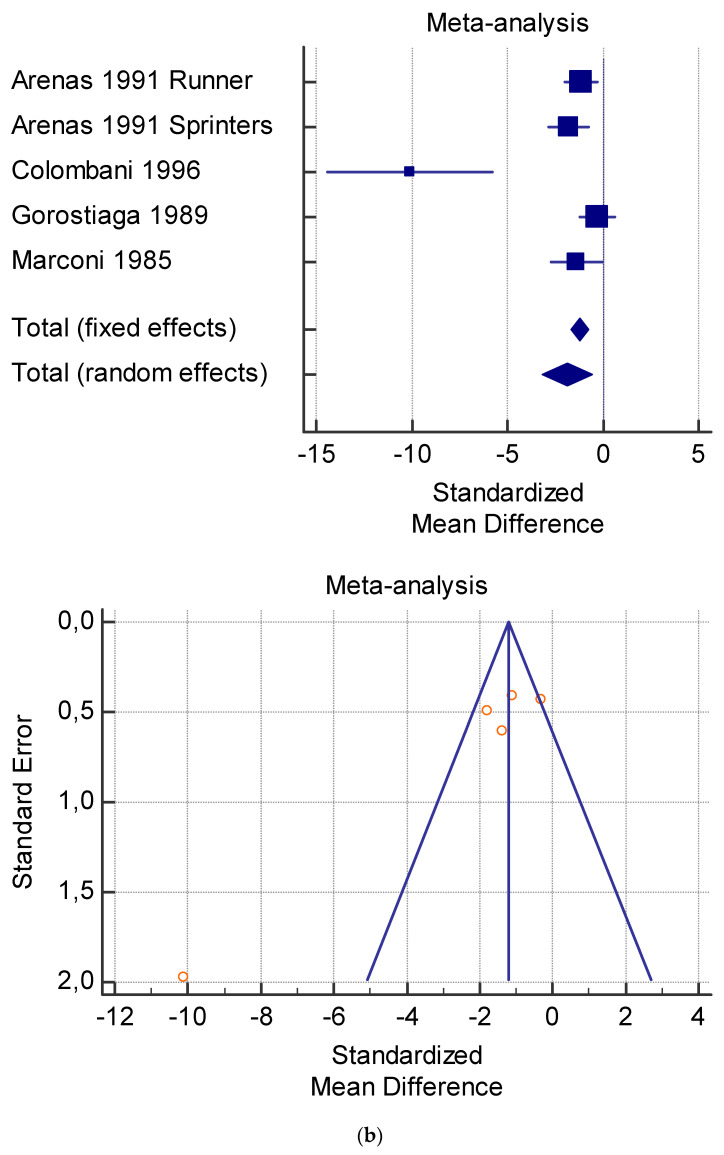

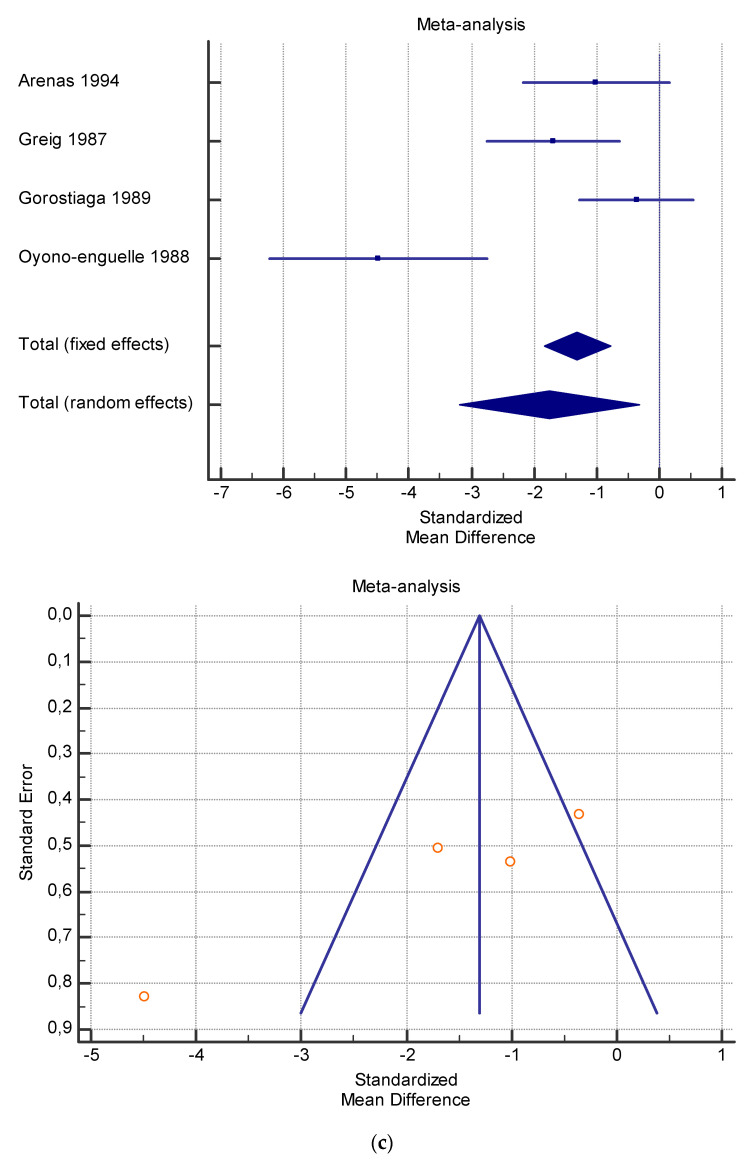

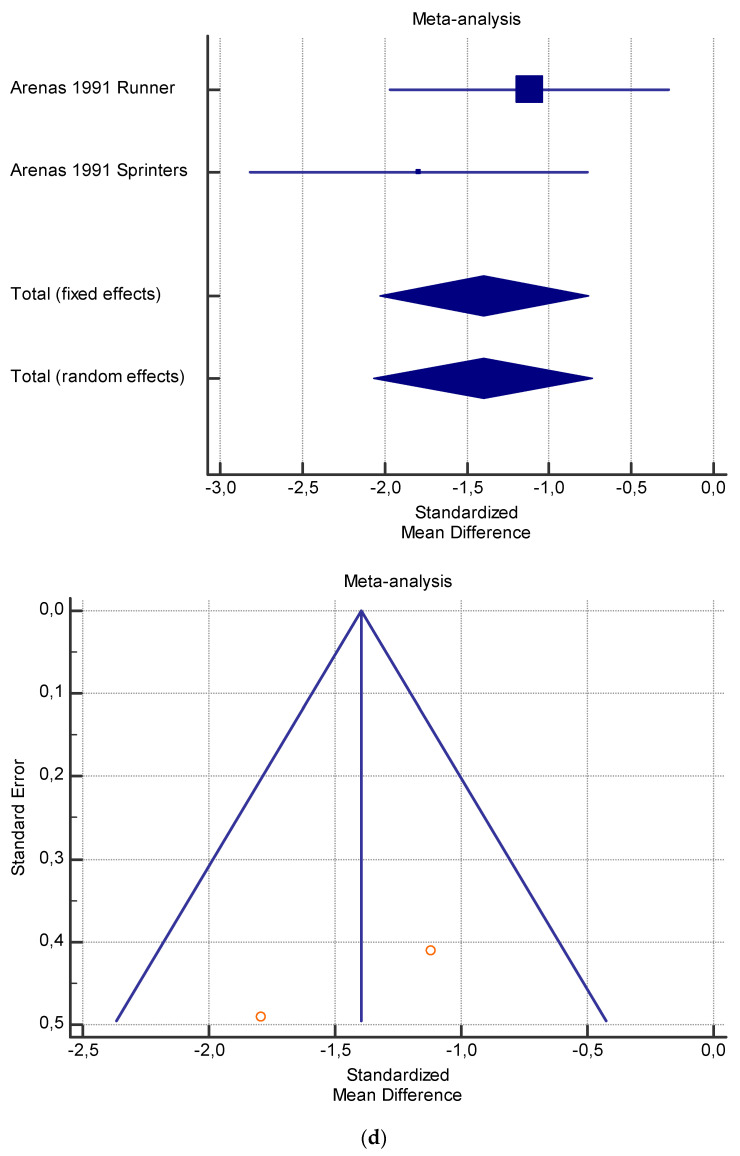
(**a**) Graphic correlation to Table 7: Serum total carnitine (µmol/L) at rest with and without the L-carnitine supplementation. (**b**) Graphic correlation to Table 7: Serum total carnitine (µmol/L) after exercise with and without the L-carnitine supplementation. (**c**) Graphic correlation to Table 7: Serum total carnitine (µmol/L) at rest after 2 g/dL for 4 weeks of L-carnitine supplementation. (**d**) Graphic correlation to Table 7: Serum total carnitine (µmol/L) after exercise after 1 g/dL for 3 weeks of L-carnitine supplementation.

**Figure 6:**
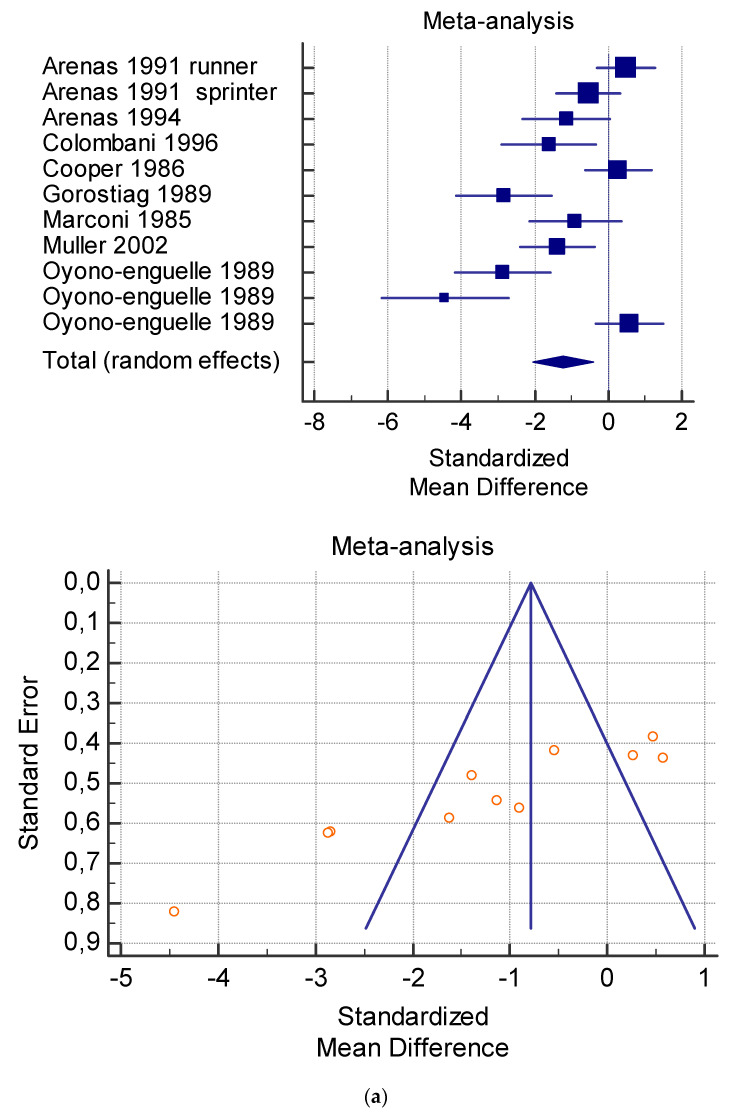

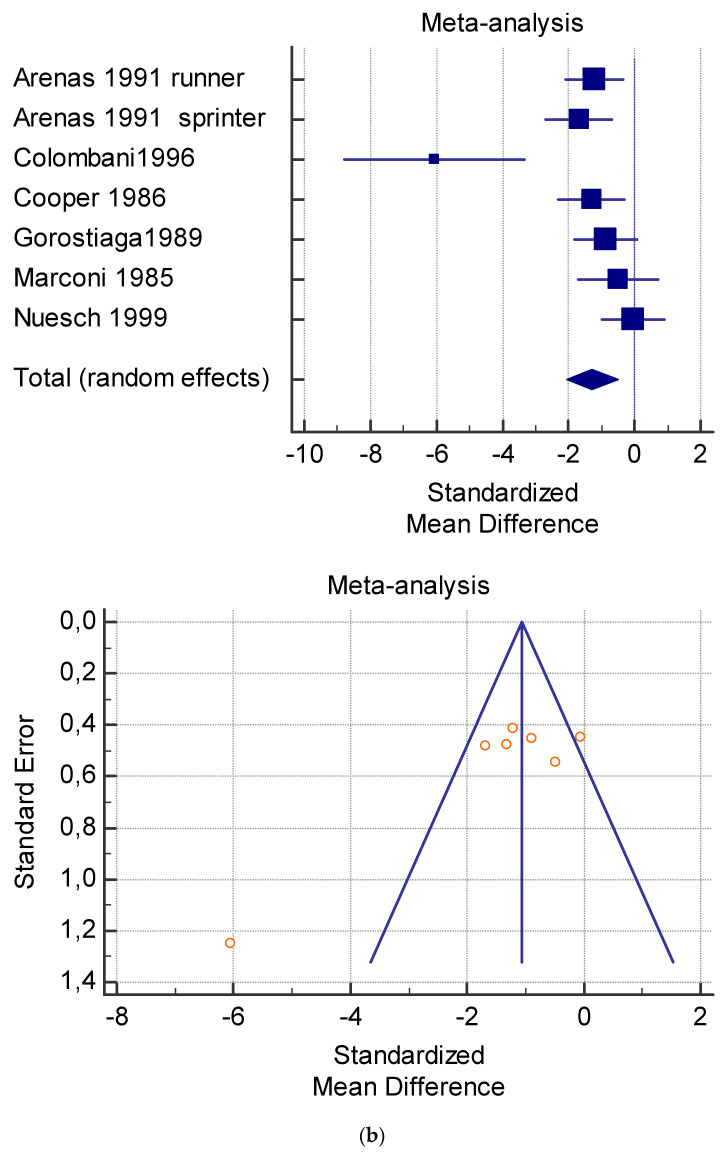

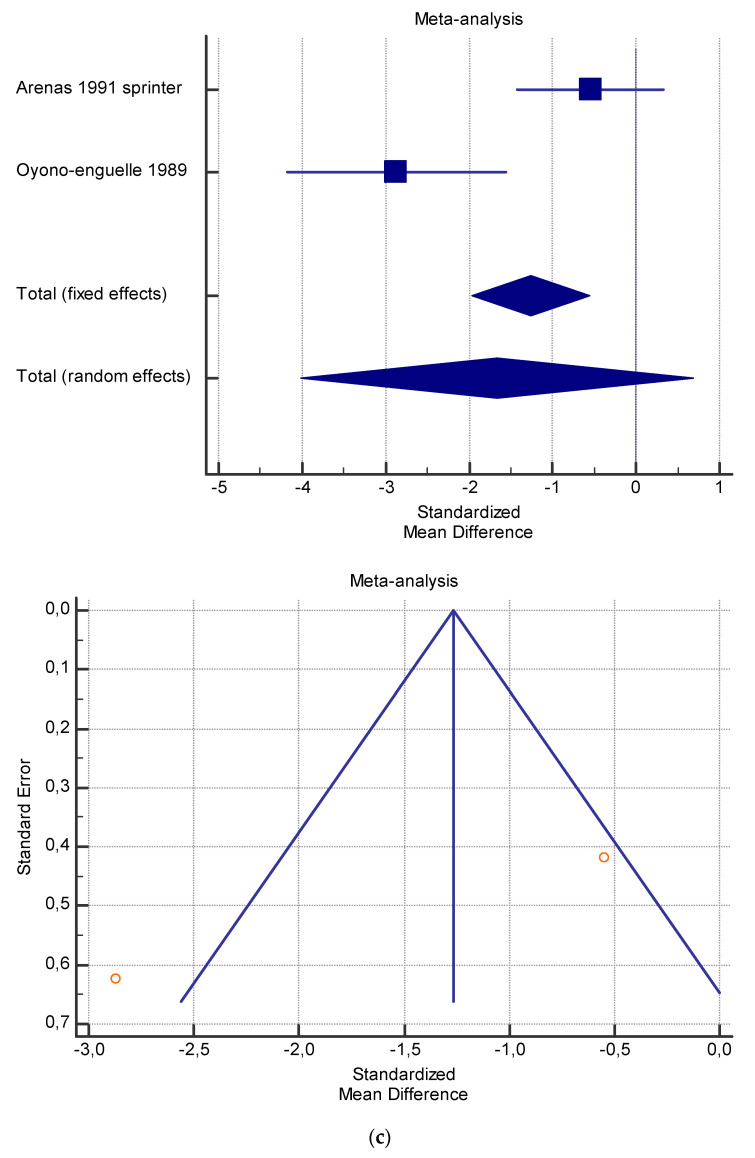

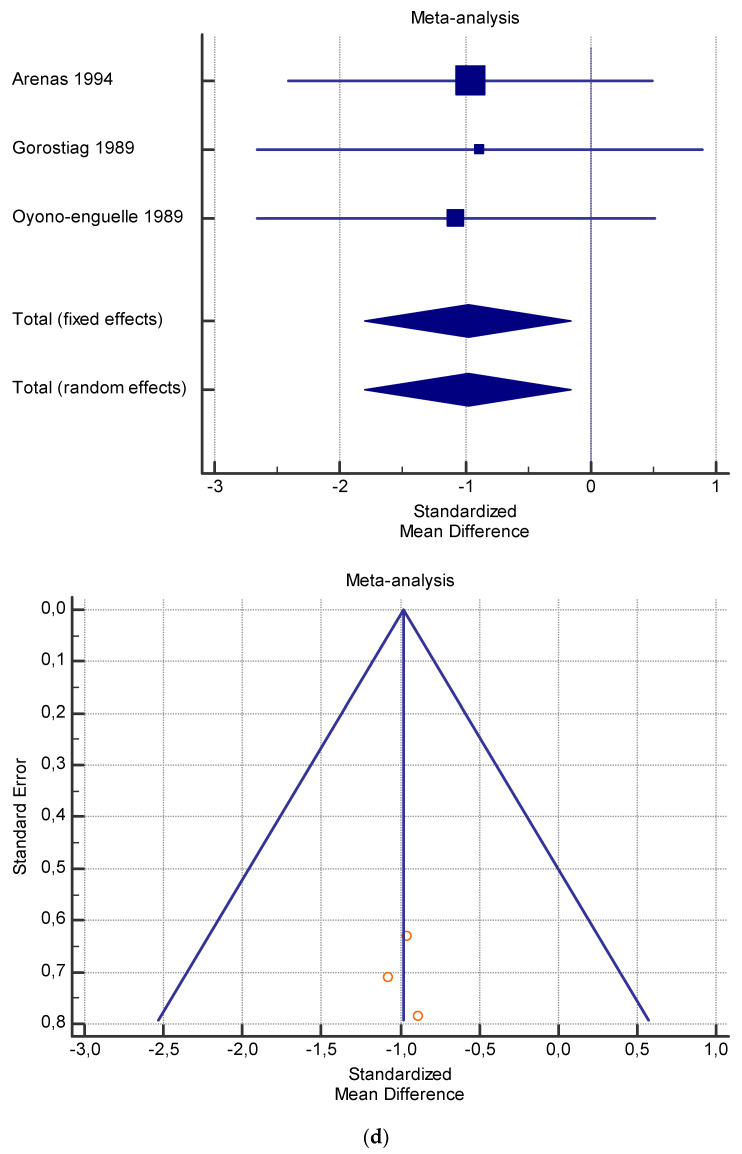
(**a**) Graphic correlation to Table 8: Serum free carnitine (µmol/L) at rest with and without the L-carnitine supplementation. (**b**) Graphic correlation to Table 8: Serum free carnitine (µmol/L) after exercise with and without the L-carnitine supplementation. (**c**) Graphic correlation to Table 8: Serum free carnitine (µmol/L) at rest after 2 g/dL for 3 weeks of L-carnitine supplementation. (**d**) Graphic correlation to Table 7: Serum.

The risk of bias assessment of the individual studies is presented in Table 1 and in the funnel plot (Figure 2, Figure 3, Figure 4, Figure 5 and Figure 6).

As shown in Table 4, Table 5, Table 6, Table 7 and Table 8, the Inconsistency test (I^2^) verified the impact of study heterogeneity on the results of the meta-analysis. The heterogeneity between studies was very low for plasma lactate at rest (I^2^ = 0.00%) (Table 4) and after exercise (I^2^ = 0.00%) (Table 5), low for VO_2_ (I^2^ = 0–36%) (Table 6) and moderate for serum carnitine (0–89%) (Table 7 and Table 8). The asymmetry between the studies, which is visible in the funnel plot (Table 4, Table 5, Table 6 and Table 7), can be explained by the heterogeneity of the sample; for this reason, publication bias and the small study effect were not significant.

### 3.5. Comparing Studies

The studies described the effects of L-carnitine supplementation on metabolic parameters, muscle features and clinical performance of healthy subjects. (Table 1). Only a few studies did not show a significant difference in muscle carnitine levels [21], muscle and plasma lactate concentration [21,22], VO_2_ [21,23,24] and physical performance [22,25].

### 3.6. Posology and Timeframe

The literature reported tests on different dosages of supplementation used over different periods of time (Table 1). The administration was made in a single day [21,22,26,27,28], 2 days [29], 5 days [30], 7 days [31,32], 10 days [33,34], 2 weeks [23,24,35], 3 weeks [36], 4 weeks [1,37,38,39,40], 6 weeks [41], 9 weeks [25], 10 weeks [42], 12 weeks [43] and 24 weeks [7].

The supplementation of L-carnitine was commonly administered orally. Only three studies used intravenous L-carnitine [21,44,45].

The effectiveness of the supplementation was not documented by all authors. Greig et al. [24] did not find any beneficial effects after 2 g for 2 and 4 weeks of oral supplementation and Brass et al. [21] did not show any modification in the content of carnitine in the muscle after a single intravenous dose of 185 μmol/kg.

According to the meta-analysis, the dose that was useful for significantly improving the level of plasma lactate was 2 g/dL for 12 weeks. The lack of sufficient data did not permit us to delineate the correct dosage to modify serum lactate and VO_2_. 

## 4. Discussion

This study shows the quantity of L-carnitine supplementation needed to obtain physical benefits and reports the metabolic markers especially related to physical activities in healthy people. The effects of the supplementation on physical performance could be applied to rehabilitation, too.

The results of our meta-analysis showed that L-carnitine supplementation had no effect on plasma lactate in individuals at rest and after exercise, neither on VO_2_ in subjects after exercise. However, the meta-analysis revealed that L-carnitine administration significantly changed serum total carnitine and serum free carnitine at rest and after exercise, as well as VO_2_ at rest.

Carnitine serum concentration correlated with changes in muscle mass and dietary intake. Moreover, a feeling of fatigue and adherence failure to rehabilitation programs and sports training could reveal values of excessive consumption. L-Carnitine supplementation could increase muscle mass, reduce body fat mass and the perception of fatigue and improve walking ability, especially in the elderly [7,46]. In addition, L-Carnitine supplementation seems to avoid the accumulation of lactate after physical exercise, a value that rises proportionally with training intensity and correlates with training endurance [29]. L-Carnitine supplementation avoids the sports related reduction of VO_2_ [26] and total and free carnitine [47]. The effectiveness of L-carnitine supplementation is also documented in a reduction of metabolic stress markers and muscle damage [35]. Thanks to these properties, dietary intake of L-carnitine could improve physical performance and increase the adherence to rehabilitation and the duration of training and rehabilitation sessions.

Moreover, the influence of physical activity on the levels of serum creatinine, VO_2_ and blood lactate makes them possible clinical markers of effectiveness of physical activities and rehabilitation training. In fact, intense physical training seems to increase acylcarnitine levels in muscles [48] and decrease free carnitine [48,49].

Where endurance exercise is concerned, no change was observed after 60 [48], 90 [50] or 225–230 min [51]. According to other studies, carnitine accumulated in muscles and was released later during recovery, based on unchanged total carnitine levels found 60 min post exercise [48] and 4–5 h after a marathon [51].

According to some authors, a single administration of L-carnitine before exercise could improve athletic performance with significant changes in free fatty acids, triacylglycerols, lactic acid [44], ability in high-intensity exercise with an increase of VO_2_ and a decrease of plasma lactate and pyruvate [26,29]. Long-term administration of L-carnitine seems to have significative effects on muscle performance, avoiding the reduction of serum total and free muscle carnitine [52] and enhancing the reduction of physical and mental fatigue and the increase of total muscle mass and serum total carnitine [7].

However, not all authors agree on the beneficial effect on muscle substrate [30], muscle carnitine level, muscle lactate accumulation, plasma lactate concentration [21,30] and changes in VO_2_ max [24,30].

Several categories of healthy people could benefit from L-carnitine supplementation: healthy adults and the elderly, overweight subjects and athletes. Healthy life expectancy seems to require a good L-carnitine status [42]. Its deficiency is related to elderly frailty; in fact, in the elderly, L-carnitine supplementation seems to increase total muscle mass [7,46] and reduce muscle fatigue [7]. The supplementation of L-carnitine has proven effective for endurance-trained athletes [38], marathon runners [33], long distance competitive walkers [23], long-distance runners and sprinters [37,52], footballers [28] and taekwondo players [32]. In athletes, dietary intake should avoid the reduction of plasma carnitine levels [38,47], and the increase of respiratory quotient [26,38] during maximal and sub-maximal exercise. Moreover, not only during maximal physical exercise [26], but also during endurance exercises, L-carnitine seems to reduce the concentration or delay the release of blood lactate, and this should improve physical performance, reducing the perception of fatigue [28]. It could decrease heart rate and positively influence aerobic capacity with an increase in running speed [41]. The supplementation should also activate lipid metabolism, facilitating the maintenance of good body weight [32,44,53,54].

### 4.1. Implication in Sports and Rehabilitation

Rehabilitation and physical activity are closely related; in fact, the former promotes the latter [55]. Thanks to this relationship, the positive effect of L-carnitine supplementation on physical performance could also be found in rehabilitation.

Heavy training [56] and high-intensity exercise [57] could cause a reduction in muscle carnitine content; thus, these two eventualities could occur in both sports and rehabilitation. This condition can worsen in the elderly, who often also have a progressive reduction in their total carnitine level linked to aging [58]. L-carnitine intake could reverse these conditions.

The health implications depend on both the carnitine- and sports-related reduction of blood lactate levels, increase of maximal oxygen consumption and fatty acid oxidation [26,59]. All these results support possible implications in rehabilitation with the increase of muscle mass and in sports, improving performance especially during high-intensity and prolonged exercise, even if not always supported by experimental evidence [60].

L-carnitine supplementation improves athletic performance with significant changes in free fatty acids, triacylglycerols, lactic acid [44], ability in high-intensity exercise with an increase of VO_2_ and a decrease of plasma lactate and pyruvate [26,29], increasing performance during endurance training by attenuating the increase in blood lactate and oxidative stress [1,25,28,29,38].

### 4.2. Ineffective Use of L-Carnitine

A few studies did not confirm the benefits of L-carnitine intake on physical performance [27], neither in muscle carnitine level [21,22], muscle and plasma lactate concentration [21,22], or respiratory exchange ratio [21,23,24]. No improvement was shown, especially after only one single administration of L-carnitine, in physical performance, respiratory exchange ratio, muscle glycogen utilization, plasma *p*-hydroxybutyrate concentration and lactic acid level [21].

## 5. Limitations 

Differences in the number of participants, as well as the other heterogeneity characteristics of the samples, may have affected the results of the present review. Moreover, despite the total number of clinical trials included in the meta-analysis, the small number of studies included in the assessment of the supplementation dosage did not allow us to evaluate the maximum amount of L-carnitine intake needed to induce changes in the metabolic markers (i.e., VO_2_ at rest, and after exercise).

## 6. Conclusions

L-carnitine supplementation is used for energetic purposes, to improve physical performance and reverse physical frailty, fatigue and weakness. This study provides a complete overview of the literature concerning the effects of L-carnitine supplementation on physical performance and rehabilitation programs in healthy subjects. Feelings of fatigue and adherence failure to rehabilitation programs and sports training could reveal values of excessive consumption. Thus, L-carnitine supplementation could be added to rehabilitation protocols in individuals whose outcomes are affected by fatigue or excessive physical stress.

Our meta-analysis showed that serum and free carnitine both at rest and after exercise and VO_2_ at rest are useful markers to follow healthy subjects during physical activity and rehabilitation programs. The dosage of supplementation that obtained a significant change in serum total carnitine was 2 g/dL for 4 weeks at rest, 1 g/dL for 3 weeks after exercise and in serum free carnitine was 2 g/dL for 3 weeks and 2 g/dL for 4 weeks at rest. Other robust trials are needed to find the best dosage to obtain positive changes in metabolic parameters and in physical performance.

## Figures and Tables

**Table 1:** The use of L-carnitine supplementation in healthy subjects: characteristics and outcomes of studies included in the systematic review according to PICOS (population, intervention, comparison, outcome and study design) criteria for the inclusion of studies.

Authors, yr	Study Design; Evidence Levels	Population, Y	Comparison Samples	Intervention: L-Carnitine Dosage	Outcomes
Marconi 1985	RT; Level 2	6 competitive walkers m 25.3 y	A: Before supplementation B: After supplementation	4 g/d, 2 w	Increased serum L-carnitine, no change in blood lactate concentrations and R at fixed workload. The 6% increase in VO_2_ was not significantly related to carnitine intake.
Cooper 1986	CT; Level 1	10 marathon runners m 19–25 y	A: Before supplementation B: After supplementation	4 g/d, 10 d	L-carnitine supplementation increased the tissue content of oxidized glutathione.
Drăgan 1987	RT; Level 2	7 athletes	A: Before supplementation B: After supplementation	1 g/d for 6 w + 2 g/d for 10 d	Carnitine group showed better obtained higher performances.
Greig 1987	CT; Level 1	19 healthy subjects, 7 m/12 f 27.1 ± 4.6 y	A: 9 s, 3 m, 6 f B: 10 s, 4 m, 6 f	A: 2 g/d, 2 w B: 2 g/d, 4 w	No change in maximum oxygen uptake (VO_2_, R).
Drăgan 1988	RT; Level 2	110 healthy subjects	A: Before supplementation B: After supplementation	1 g/d for 3 w	Improved athletic performance, lower lactic acid.
Oyono-Enguelle 1988	RT; Level 2	10 exercising subjects -	A: Before supplementation B: After supplementation	2 g/d, 4 w	After L-carnitine intake, the levels returned to their initial values 6–8 w after cessation of the supply.
Soop 1988	RT; Level 2	7 moderately trained subjects 19–31 y	A: Before supplementation B: After supplementation	5 g/d, 5 d	No change in O2 uptake and arterial levels and turnover of FFA after L-carnitine supplementation.
Gorostiaga 1989	RT; Level 2	10 trained athletes, 9 m/1 f 25.8 ± 2.2 y	A: Before supplementation B: After supplementation	2 g/d, 4 w	Reduced R during submaximal exercise after supplementation. Increased lipid use by muscles during exercise. Oxygen uptake, heart rate, blood glycerol and resting plasma free fatty acid concentrations presented a nonsignificant trend.
Siliprandi 1990	CT; Level 1	10 moderately trained subjects 23–30 y	A: Before supplementation B: After supplementation	2 g/d, 2 d	L-carnitine supplementation decreased plasma lactate and pyruvate concentration.
Vecchiet 1990	CT; Level 1	10 moderately trained subjects 22–30 y	A: Before supplementation B: After supplementation	2 g/d, one single dosage	Increased VO_2_, maximal oxygen uptake, power output and reduced pulmonary ventilation and plasma lactate after L-carnitine supplementation.
Wyss 1990	RT; Level 2	7 healthy subjects 22.2 ± 2.3 y	A: Before supplementation B: After supplementation	3 g, 1 w	Decreased R, and rate of carbohydrate transformation during hypoxia after L-carnitine supplementation.
Arenas 1991	CT; Level 1	24 athletes 19–27 y	A: 13 and 11 s placebo B: 11 s sprinters L-carnitine C: 13 long-distance runners	1 g/d, 24 w	The supplementation avoids the decrease of total and free muscle carnitine due to training in athletes.
Huertas 1992	CT; Level 1	14 athletes	A: Before supplementation B: After supplementation	2 g/d, 4 w	Increase in respiratory-chain enzyme activities in the muscle.
Natali 1993	CT; Level 1	20 healthy subjects A: 29.5 ± 1.7 years B: 29 ± 2 y	A: 8 healthy m B: 12 healthy	A: 1 g + 0.5 g/h iv B: 3 g, iv. 40′ before exercise	No changes during exercise with L-carnitine intake, but increased fatty acid oxidation during recovery.
Arenas 1994	CT; Level 1	16 long-distance runners 28.3 ± 7.1	A: 8 s placebo B: 8 s L-carnitine	2 g/d, 4 w	The supplementation increased pyruvate dehydrogenase complex activities.
Brass 1994	CT; Level 1	14 athletes 23–40 y	A: Before supplementation B: After supplementation	185 μmol/kg/1 d iv	No effect on skeletal muscle carnitine homeostasis during exercise: on R, muscle lactate accumulation, plasma lactate concentration, muscle glycogen utilization, plasma *p*-hydroxybutyrate concentration after L-supplementation.
Colombani 1996	CT; Level 1	7 m runners 36 ± 3 y	A: Before supplementation B: After supplementation	4 g/d, 1 d	No effect on performance, and no changes in running time and in R after L-carnitine supplementation.
Giamberardino 1996	RT; Level 2	6 healthy untrained subjects, 26 ± 3.8 y	A: Before supplementation B: After supplementation	A: 3 g/d, 3 w	Protective effect against pain and damage.
Swart 1997	RT; Level 2	7 marathon runners -	A: Before supplementation B: After supplementation	2 g/d, 6 w	After L-carnitine supplementation: increase of peak treadmill running speed of 5.68%, average VO_2_, free carnitine levels, decreased heart rate and respiratory exchange ratio values.
Nuesch 1999	RT; Level 2	9 athletes	A: Before supplementation B: After supplementation	1 g/d after treadmill	In athletes without L-carnitine intake, plasma free carnitine concentration decreased significantly 10 min after exercise compared with baseline. In athletes with oral L-carnitine supplementation, the elevated plasma concentration of free carnitine at baseline did not decrease after maximal exercise.
Muller 2002	RT; Level 2	10 healthy untrained subjects 5 m/5 f 36.4 ± 12.8 y	A: Before supplementation B: After supplementation	3 g/d, 10 d	Significant increase in fatty acid oxidation, muscle weight, total body water and metabolic rate.
Wachter 2002	RT; Level 2	8 healthy subjects 23–25 y	A: Before supplementation B: After supplementation	4 g/d, 12 w	Significant increase in physical performance after L-carnitine supplementation.
Stuessi 2005	CT; Level 1	12 m athletes, 25 ± 3 y	A: Before supplementation B: After supplementation	2 g/d, lump sum	No enhanced performance in endurance after L-carnitine supplementation.
Malaguarnera 2007	CT; Level 1	64 healthy elderly subjects A: 101 ± 1.3 y B: 101 ± 1.4 y	A: 32 s L-carnitine B: 34 s placebo	2 g/d, 24 w	Improvements in fat mass, muscle mass, blood carnitine, acylcarnitine, physical and mental fatigue.
Chun 2011	RT; Level 2	36 m soccer players 20.67± 1.21 y	A: 6 s, 2g L-carnitine B: 6 s, 3g L-carnitine, C: 6 s, 4g L-carnitine, D: 6 s,5g L-carnitine E: 6 s, 6g L-carnitine F: 6 s, no intake	2–6 g/d, 4 w	L-carnitine can enhance endurance and recovery from fatigue in athletes, increase VO_2_ and decrease lactate concentration
Orer 2014	CT; Level 1	26 footballers 18.4 ±0.5 y	A: 12 athletes placebo B: 14 athletes L-carnitine	A; 3 g/d, 1 d B: 4 g/d, 1 d	Supplementation affected performance positively in terms of running speed corresponding to specific lactate concentrations, lactic acid and Borg scale responses corresponding to running speeds.
Parandak 2014	CT; Level 1	21 healthy subjects A: 22.2 ± 1.1 y B: 22.0 ± 1.0 y	A: 10 L-carnitine B: 11 placebo	2 g/d, 2 w	TAC increased significantly 14 days after supplementation. Serum MDA-TBARS, CK, TAC, LDH were significantly lower 24 h after exercise. The supplementation alleviated the effects on lipid peroxidation and muscle damage markers.
Bradasawi 2016	CT; Level 1	50 healthy subjects A: 68.2 ± 6.3 y B: 68.2 ± 6.5 y	A: 26 s L-carnitine B: 24 s placebo	1.5 g/d, 10 w	No significant changes in free L-carnitine, total L-carnitine, acyl L-carnitine blood level and frailty biomarkers (IL-6, TNF-α, and IGF-1) between the placebo group and carnitine group.
Koozehchian 2018	CT; Level 1	23 trained subjects A: 24.5 ± 1.5 y B: 25.5 ±1.5 y	A: 11 s placebo B: l2 s L-carnitine	2 g/d, 9 w	No significant influence on muscle mass though upper/lower body strength improved.
Mor 2018	CT; Level 1	16 taekwondo players 18–28 y	A: 8 s L-carnitine B: 8 s placebo	1 g/d, 1 w	Low body fat mass.

CT: Clinical trial, RT: Retrospective study, PS: Prospective study, s: Subjects, y: Years old, A: Group 1, B: Group 2, g: Gram, d: Days, w: Week, m: Months, h: Hours, IL: Interleukin, TNF: Tumor necrosis factor, ILGF: Insulin-like growth factor, FIS: Frailty Index score, PASE: Physical Activity Scale for Elderly, WST: walking speed test, 2MST: 2-min step test, TUG: time up and go test, CST: chair stand test, RPT: rapid pace test, SST: shoulder strength test, PEFR: peak expiratory flow rate, ADL: Activities of daily living, IADL: Instrumental activities of daily living, RT: Resistance training, VO_2_: maximum oxygen uptake, FFA: Free Fatty Acids, h: Hour, MRI: magnetic resonance imaging, R: respiratory exchange ratio, m: males, f: females, MMSE: Mini-Mental State Examination, TAC: Plasma total antioxidant capacity, MDA: malondialdehyde, TBARS: thiobarbituric acid-reactive substance, CK: creatine kinase, LDH: lactate dehydrogenase, S: Supplementation, TMAO: trimethylamine-N-oxide, 3IGF-BP3: Serum insulin-like growth factor-binding protein, iv: Intravenously.

**Table 2:** Risk of bias summary for each included study.

Study, Year	Random Sequence Generation	Allocation Concealment	Blinding Participants	Blinding of Outcome Assessment	Incomplete Data	Selective Reporting	Other Bias	Risk of Bias
Arenas 1991	+	+	+	+	+	+	+	Low risk
Arenas 1994	+	+	+	+	+	+	+	Low risk
Bradasawi 2016	+	+	+	+	+	+	+	Low risk
Brass 1994	+	+	+	+	+	+	+	Low risk
Colombani 1996	+	+	+	+	+	+	+	Low risk
Chun 2011	+	+	-	-	+	+	+	Low risk
Cooper 1986	-	-	-	-	+	+	-	High risk
Dragan 1987	+	+	+	+	+	+	+	Low risk
Dragan 1988	+	+	+	+	+	+	+	Low risk
Giamberardino 1996	+	+	+	+	+	+	+	Low risk
Gorostiaga 1989	-	-	+	+	+	+	+	Low risk
Greig 1987	+	+	+	+	+	+	+	Low risk
Huertas 1992	+	+	+	+	+	+	+	Low risk
Koozehchian 2018	+	+	+	+	+	+	+	Low risk
Malaguarnera 2007	+	+	+	+	+	+	+	Low risk
Marconi 1985	+	+	-	-	+	+	+	Low risk
Mor 2018	+	+	+	+	+	+	+	Low risk
Muller 2002	-	-	-	-	+	+	+	High risk
Natali 1993	+	+	-	-	+	+	+	Low risk
Nuesch et al.1999	-	-	-	-	+	+	+	High risk
Orer 2014	+	+	+	+	+	+	+	Low risk
Oyono-Enguelle 1988	-	-	-	-	+	+	+	High risk
Parandak 2014	+	+	+	+	+	+	+	Low risk
Siliprandi 1990	+	+	+	+	+	+	+	Low risk
Soop 1988	-	-	+	+	+	+	+	Low risk
Stuessi 2005	+	+	+	+	+	+	+	Low risk
Swart 1997	-	-	-	-	+	+	+	High risk
Vecchiet 1990	+	+	+	+	+	+	+	Low risk
Wachter 2002	-	-	-	-	+	+	+	High risk
Wyss 1990	-	-	+	+	+	+	+	High risk

+: low risk of bias; -: high risk of bias.

**Table 3:** GRADE quality of evidence.

Quality Assessment					Summary of Findings		Quality of Evidence GRADE
N° of studies	Limitations	Inconsistency	Indirectness	Publication bias	Characteristics of	IRR (95% CI)	
30 studies	No significant limitations	No serious inconsistency	No serious indirectness	Unlikely	Population: Adults Intervention: L-carnitine intake Comparison: - Before and after intake - L-carnitine vs. placebo Outcomes: Improvement in physical performance	Fixed and Random effects model:Table 4, Table 5, Table 6 and Table 7	Moderate-High

**Table 4:** Plasma lactate (mmol/L) at rest with and without L-carnitine supplementation.

Authors	N 1	N 2	Carnitine Dosage	Mean 1	SD 1	Mean 2	SD 2	SMD	SE	95% CI	t	*p*	Weight (%)		Test for Heterogeneity	
													Fixed	Random		
Colombani 1996	7	7	4 g/d. 1 d	1.3	0.1	1.4	0.1	−0.936	0.531	−2.092 to 0.220			7.93	7.93	Q	8.0319
Chun 2011	6	6	2 g/d. 4 w	1.88	0.46	1.78	0.28	0.242	0.535	−0.950 to 1.434			7.80	7.80	DF	10
Chun 2011	6	6	3 g/d. 4 w	1.41	0.80	1.04	0.49	0.515	0.543	−0.695 to 1.725			7.57	7.57	Significance level	*p* = 0.6257
Chun 2011	6	6	4 g/d. 4 w	1.10	0.23	1.29	0.36	−0.580	0.546	−1.796 to 0.636			7.49	7.49	I^2^ (inconsistency)	0.00%
Chun 2011	6	6	5 g/d. 4 w	1.38	0.57	1.50	0.79	−0.161	0.534	−1.350 to 1.029			7.83	7.83	95% CI for I^2^	0.00 to 50.67
Chun 2011	6	6	6 g/d. 4 w	1.38	0.47	1.31	0.25	0.172	0.534	−1.018 to 1.361			7.83	7.83		
Greig 1987	9	9	2 g/d. 2 w	1.04	0.66	1.14	0.28	−0.188	0.450	−1.142 to 0.766			11.02	11.02		
Greig 1987	10	10	2 g/d. 4 w	0.8	0.3	0.9	0.3	−0.319	0.431	−1.225 to 0.587			12.00	12.00		
Oyono-Enguelle 1988	10	10	2 g/d. 4 w	0.93	0.35	0.86	0.34	0.194	0.429	−0.708 to 1.096			12.11	12.11		
Watcher 2002	8	8	4 g/d. 12 w	0.9	0.1	0.8	0.2	0.598	0.484	−0.441 to 1.637			9.52	9.52		
Wyss 1990	7	7	3 g/d. 1 w	1.5	0.7	1.4	0.7	0.134	0.501	−0.958 to 1.225			8.90	8.90		
Total (fixed effects)	81	81						−0.0257	0.149	−0.321 to 0.269	−0.172	0.864	100.00	100.00		
Total (random effects)	81	81						−0.0257	0.149	−0.321 to 0.269	−0.172	0.864	100.00	100.00		
Chun 2011	6	6	2 g/d. 4 w	1.88	0.46	1.78	0.28	0.242	0.535	−0.950 to 1.434			24.44	24.44	*Q*	0.9555
Greig 1987	10	10	2 g/d. 4 w	0.8	0.3	0.9	0.3	−0.319	0.431	−1.225 to 0.587			37.62	37.62	*DF*	2
Oyono-Enguelle 1988	10	10	2 g/d. 4 w	0.93	0.35	0.86	0.34	0.194	0.429	−0.708 to 1.096			37.94	37.94	Significance level	*p* = 0.6202
Total (fixed effects)	26	26						0.0129	0.264	−0.518 to 0.544	0.0486	0.961	100.00	100.00	I^2^	0.00%
Total (random effects)	26	26						0.0129	0.264	−0.518 to 0.544	0.0486	0.961	100.00	100.00	95% CI for I^2^	0.00 to 92.98

Mm: Millimoles, l: liter, d: day; L-carn: L-carnitine, N: number of subjects, 1: subjects that did not use supplementation, 2: subjects used L-carnitine, g: grams, w: weeks, SMD: standard mean differences, SE: standard error, CI: confidential intervals, SD: standard deviation, I^2^: inconsistency.

**Table 5:** Plasma lactate (mmol/L) after exercises with and without L-carnitine supplementation.

Authors	N 1	N 2	Carn Dosage	Training	Mean 1	SD 1	Mean 2	SD 2	SMD	SE	95% CI	t	*p*	Weight (%)		Test for Heterogeneity	
														Fixed	Random		
Colombani 1996	7	7	4 g/d. 1 d	20 km of running	1.3	0.1	1.40	0.10	−0.936	0.531	−2.092 to 0.220			7.11	7.11	Q	10.937
Chun 2011	6	6	2 g/d. 4 w	After exercise	7.02	0.52	6.88	0.68	0.213	0.535	−0.978 to 1.404			7.01	7.01	DF	11
Chun 2011	6	6	3 g/d. 4 w	After exercise	6.39	1.64	6.25	1.05	0.0938	0.533	−1.094 to 1.282			7.05	7.05	Significance level	*p* = 0.448
Chun 2011	6	6	4 g/d. 4 w	After exercise	6.80	2.09	5.87	1.08	0.516	0.543	−0.694 to 1.726			6.79	6.79	I^2^ (inconsistency)	0.00%
Chun 2011	6	6	5 g/d. 4 w	After exercise	6.96	1.87	6.31	1.56	0.348	0.537	−0.849 to 1.546			6.93	6.93	95% CI for I^2^	0.00 to 58.20
Chun 2011	6	6	6 g/d. 4 w	After exercise	6.80	0.98	6.09	1.16	0.610	0.547	−0.609 to 1.829			6.69	6.69		
Greig 1987	9	9	2 g/d. 2 w	After 30′ of cycling	11.7	2.1	10.10	2.60	0.645	0.462	−0.334 to 1.623			9.40	9.40		
Greig 1987	10	10	2 g/d. 4 w	After 30′ of cycling	11.8	4.4	10.70	3.00	0.280	0.431	−0.625 to 1.184			10.80	10.80		
Koozehchian2018	11	11	2 g/d. 9 w	Maximum exercise	5.73	1.14	4.60	0.97	1.027	0.438	0.112 to 1.942			10.42	10.42		
Oyono-Enguelle 1988	10	10	2 g/d. 4 w	After 60′ cycling	1.72	0.62	1.68	0.56	0.0648	0.428	−0.835 to 0.965			10.91	10.91		
Watcher 2002	8	8	4 g/d. 12 w	Power exercise	9.3	0.4	9.20	1.20	0.106	0.473	−0.909 to 1.120			8.95	8.95		
Wyss 1990	7	7	3 g/d. 1 w	Maximum exercise	8.1	1.7	8.80	3.30	−0.250	0.503	−1.344 to 0.845			7.93	7.93		
Total (fixed effects)	92	92							0.250	0.142	−0.0297 to 0.529	1.764	0.079	100.00	100.00		
Total (random effects)	92	92							0.250	0.142	−0.0297 to 0.529	1.764	0.079	100.00	100.00		
Authors	N 1	N 2	Carn. Dosage	Training	Mean 1	SD 1	Mean 2	SD 2	SMD	SE	95% CI	t	*p*	Weight (%)		Test of heterogeneity	
														Fixed	Random	Q	0.1252
Greig 1987	10	10	2 g/d. 4 w	After 30′ of cycling	11.8	4.4	10.70	3.00	0.280	0.431	−0.625 to 1.184			49.75	49.75	DF	1
Oyono-Enguelle 1988	10	10	2 g/d. 4 w	After 60′ cycling	1.72	0.62	1.68	0.56	0.0648	0.428	−0.835 to 0.965			50.25	50.25	Significance level	*p* = 0.7235
Total (fixed effects)	20	20							0.172	0.304	−0.443 to 0.787	0.566	0.575	100.00	100.00	I^2^	0.00%
Total (random effects)	20	20							0.172	0.304	−0.443 to 0.787	0.566	0.575	100.00	100.00	95% CI for I^2^	0.00 to 0.00

Mm: Millimoles, l: Liter, d: Day, w: weeks; L-carn: L-carnitine, N: number of subjects, 1: subjects that did not use carn, 2: subjects used carn, g: Grams, SMD: standard mean differences, SE: standard error, CI: confidential intervals, SD: standard deviation, I^2^: inconsistency.

**Table 6:** Maximal oxygen consumption VO_2_ (mL/min/kg) at rest with and without L-carnitine supplementation and after exercise with and without L-carnitine supplementation.

Authors	Carnitine Dosage	N1	N2	Mean 1	SD 1	Mean 2	SD 2	SMD	SE	95% CI	t	*p*	Weight (%)		Test for Heterogeneity	
													Fixed	Random		
Chun 2011	2 g/d, 4 w	6	6	57.07	3.07	57.52	3.42	−0.128	0.533	−1.316 to 1.061			7.72	7.72	Q	6.3892
Chun 2011	3 g/d, 4 w	6	6	62.06	7.29	60.00	6.16	0.282	0.536	−0.912 to 1.476			7.65	7.65	DF	10
Chun 2011	4 g/d, 4 w	6	6	59.33	7.57	67.26	7.66	−0.961	0.568	−2.226 to 0.304			6.82	6.82	Significance level	*p* = 0.7816
Chun 2011	5 g/d, 4 w	6	6	59.78	6.47	63.64	4.86	−0.622	0.548	−1.843 to 0.598			7.32	7.32	I^2^ (inconsistency)	0.00%
Chun 2011	6 g/d, 4 w	6	6	59.90	4.88	61.20	4.44	−0.257	0.535	−1.450 to 0.936			7.67	7.67	95% CI for I^2^	0.00 to 37.99
Greig 1987	2 g/d, 2 w	9	9	41.4	7.5	41.6	8.8	−0.0233	0.449	−0.975 to 0.928			10.90	10.90		
Greig 1987	2 g/d, 4 w	10	10	45.2	12.0	43.6	12.0	0.128	0.429	−0.773 to 1.028			11.95	11.95		
Marconi 1985	4 g/d, 2 w	6	6	54.5	3.7	57.8	4.7	−0.720	0.553	−1.951 to 0.511			7.19	7.19		
Parandak 2014	2 g/d, 2 w	11	10	22.4	0.8	23.0	0.6	−0.809	0.438	−1.724 to 0.107			11.47	11.47		
Vecchiet 1990	2 g/d, one time	10	10	43.91	7.87	47.18	9.59	−0.357	0.432	−1.265 to 0.551			11.77	11.77		
Wachter 2002	4 g/d, 12 w	8	8	17.1	1.6	18.0	1.9	−0.484	0.480	−1.515 to 0.546			9.52	9.52		
Total (fixed effects)		83	83					−0.339	0.148	−0.632 to −0.0465	−2.288	0.023	100.00	100.00		
Total (random effects)		83	83					−0.339	0.148	−0.632 to −0.0465	−2.288	0.023	100.00	100.00		
**Authors**	**Carnitine Dosage**	**N1**	**N2**	**Mean 1**	**SD 1**	**Mean 2**	**SD 2**	**SMD**	**SE**	**95% CI**	**t**	***p***	**Weight (%)**		**Test for Heterogeneity**	
													Fixed	Random		
Greig 1987	2 g/d, 2 w	9	9	41.4	7.5	41.6	8.8	−0.0233	0.449	−0.975 to 0.928			48.72	49.19	Q	1.5691
Parandak 2014	2 g/d, 2 w	11	10	22.4	0.8	23.0	0.6	−0.809	0.438	−1.724 to 0.107			51.28	50.81	DF	1
Total (fixed effects)		20	20					−0.426	0.313	−1.061 to 0.209	−1.359	0.182	100.0	100.00	Significance level	*p* = 0.2103
Total (random effects)		20	20					−0.422	0.393	−1.218 to 0.373	−1.076	0.289	100.0	100.00	I^2^	36.27%
															95% CI for I^2^	0.00 to 0.00
Chun 2011	2 g/d, 4 w	6	6	57.07	3.07	57.52	3.42	−0.128	0.533	−1.316 to 1.061			39.25	39.25	Q	0.1393
Greig 1987	2 g/d, 4 w	10	10	45.2	12.0	43.6	12.0	0.128	0.429	−0.773 to 1.028			60.75	60.75	DF	1
Total (fixed effects)		16	16					0.0274	0.334	−0.655 to 0.710	0.0820	0.935	100.0	100.00	Significance level	*p* = 0.7089
Total (random effects)		16	16					0.0274	0.334	−0.655 to 0.710	0.0820	0.935	100.0	100.00	I^2^	0.00%
															95% CI for I^2^	0.00 to 0.00

VO_2_: Maximal oxygen consumption, w: weeks, g: grams, d: days, l: milliliter, min: minutes, L-carn: L-carnitine, SD: standard deviation, N: number of subjects, 1: subjects that did not use carnitine, 2: subjects used carn, SMD: standard mean differences, SE: standard error, CI: confidential intervals, SD: standard deviation, I^2^: inconsistency.

**Table 7:** Serum total carnitine (µmol/L) at rest with and without L-carnitine supplementation and after exercise with and without L-carnitine supplementation.

Authors	Carnitine Dosage		N1	N2	Mean 1	SD 1	Mean 2	SD 2	SMD	SE	95% CI	t	*p*	Weight (%)		Test for Heterogeneity	
														Fixed	Random		
Arenas 1991 Runner	1 g/d, 3 w		13	13	28	2	26.3	4.134	0.510	0.386	−0.287 to 1.308			13.57	8.60	Q	62.7138
Arenas 1991 Sprinters	1 g/d, 3 w		11	11	22.8	1.8	22.5	1.4	0.179	0.411	−0.678 to 1.036			11.99	8.47	DF	12
Arenas 1994	2 g/d, 4 w		7	7	30.9	3.8	34.8	3.4	−1.012	0.536	−2.179 to 0.155			7.06	7.76	Significance level	*p* < 0.0001
Colombani 1996	4 g/d, 1 d		7	7	51.4	2.8	57.9	1.6	−2.668	0.710	−4.215 to −1.120			4.02	6.73	I^2^ (inconsistency)	80.87%
Gorostiaga 1989	2 g/d, 4 w		10	10	44.5	4.5	46.1	4.0	−0.360	0.432	−1.268 to 0.548			10.86	8.35	95% CI for I^2^	68.26 to 88.47
Greig 1987	2 g/d, 2 w		9	9	55.3	7.6	78.9	16.3	−1.767	0.537	−2.905 to −0.629			7.03	7.75		
Greig 1987	2 g/d, 4 w		10	10	41.3	8.4	56.0	8.2	−1.696	0.505	−2.758 to −0.634			7.94	7.94		
Marconi 1985	4 g/d, 2 w		6	6	64.3	2.1	86.8	17.1	−1.704	0.636	−3.122 to −0.287			5.00	7.16		
Muller 2002	3 g/d, 10 d		10	10	47.07	6.82	59.86	9.52	−1.479	0.488	−2.504 to −0.454			8.51	8.04		
Oyono-Eguelle 1988	2 g/d, 3 w		10	10	62.0	3.8	72.9	4.3	−2.572	0.591	−3.813 to −1.332			5.81	7.44		
Oyono-Enguelle 1988	2 g/d, 4 w		10	10	62.0	3.8	79.8	3.8	−4.486	0.829	−6.227 to −2.745			2.95	6.05		
Oyono-Enguelle 1988	2 g/d, 12 w		10	10	62.0	3.8	67.4	3.8	−1.361	0.479	−2.368 to −0.354			8.82	8.09		
Swart 1997	2 g/d, 6 w		7	7	52.9	5.1	61.93	7.3	−1.342	0.561	−2.564 to −0.120			6.44	7.61		
Total (fixed effects)			120	120					−1.085	0.142	−1.366 to −0.805	−7.624	<0.001	100.00	100.00		
Total (random effects)			120	120					−1.417	0.331	−2.069 to −0.764	−4.277	<0.001	100.00	100.00		
**Authors**	**Carn. Dosage**	**Training**	**N1**	**N2**	**Mean 1**	**SD 1**	**Mean 2**	**SD 2**	**SMD**	**SE**	**95% CI**	**t**	***p***	**Weight (%)**		**Test for Heterogeneity**	
Arenas 1991 Runner	1 g/d, 3 w	After exercise	13	13	26.3	1.9	29.0	2.7	−1.120	0.410	−1.967 to −0.273			32.13	23.93	Q	26.2778
Arenas 1991 Sprinters	1 g/d, 3 w	After exercise	11	11	21.6	1.5	24.3	1.4	−1.790	0.491	−2.814 to −0.766			22.44	23.00	DF	4
Colombani 1996	4 g/d, 1 d	After running	7	7	57.9	1.6	122.4	8.3	−10.100	1.973	−14.399 to −5.801			1.39	7.79	Significance level	84.78
Gorostiaga 1989	2 g/d, 4 w	After 40′ of exercise	10	10	47.4	5.9	49.1	4.9	−0.300	0.431	−1.205 to 0.605			29.14	23.70	I^2^ (inconsistency)	84.78%
Marconi 1985	4 g/d, 2 w	After 120′ of treadmill	6	6	86.8	17.1	109.8	13.4	−1.382	0.603	−2.725 to −0.0385			14.89	21.59	95% CI for I^2^	66.07 to 93.17
Total (fixed effects)			47	47					−1.195	0.233	−1.657 to −0.733	−5.138	<0.001	100.00	100.00		
Total (random effects)			47	47					−1.918	−1.836	−3.139 to −0.533	−2.798	0.006	100.00	100.00		
**Authors**	**Carnitine Dosage**		**N1**	**N2**	**Mean 1**	**SD 1**	**Mean 2**	**SD 2**	**SMD**	**SE**	**95% CI**	**t**	***p***	**Weight (%)**		**Test for Heterogeneity**	
														Fixed	Random		
Arenas 1994	2 g/d, 4 w		7	7	30.9	3.8	34.8	3.4	−1.012	0.536	−2.179 to 0.155			24.52	25.62	Q	20.4203
Greig 1987	2 g/d, 4 w		10	10	41.3	8.4	56.0	8.2	−1.696	0.505	−2.758 to −0.634			27.55	26.03	DF	3
Gorostiaga 1989	2 g/d, 4 w		10	10	44.5	4.5	46.1	4.0	−0.360	0.432	−1.268 to 0.548			37.68	26.97	Significance level	*p* = 0.0001
Oyono-Enguelle 1988	2 g/d, 4 w		10	10	62.0	3.8	79.8	3.8	−4.486	0.829	−6.227 to −2.745			10.25	21.37	I^2^	85.31%
Total (fixed effects)			37	37					−1.311	0.265	−1.839 to −0.782	−4.942	<0.001	100.00	100.00	95% CI for I^2^	63.69 to 94.06
Total (random effects)			37	37					−1.757	0.718	−3.187 to −0.326	−2.448	0.017	100.00	100.00		
**Authors**	**Carn. Dosage**	**Training**	**N1**	**N2**	**Mean 1**	**SD 1**	**Mean 2**	**SD 2**	**SMD**	**SE**	**95% CI**	**t**	***p***	**Weight (%)**		**Test for Heterogeneity**	
														Fixed	Random		
Arenas 1991 Runners	1 g/d, 3 w	After exercise	13	13	26.3	1.9	29.0	2.7	−1.120	0.410	−1.967 to −0.273			58.88	58.09	Q	1.0969
Arenas 1991 Sprinters	1 g/d, 3 w	After exercise	11	11	21.6	1.5	24.3	1.4	−1.790	0.491	−2.814 to −0.766			41.12	41.91	DF	1
Total (fixed effects)			24	24					−1.396	0.315	−2.029 to −0.762	−4.432	<0.001	100.00	100.00	Significance level	*p* = 0.2949
Total (random effects)			24	24					−1.401	0.331	−2.066 to −0.735	−4.236	<0.001	100.00	100.00	I^2^	8.83%
																95% CI for I^2^	0.00 to 0.00

w: weeks, g: grams, d: days, l: milliliter, min: minutes; L-carn: L-carnitine, SD: standard deviation, N: number of subjects, 1: subjects that did not use carn.; 2: subjects used carnitine, SMD: standard mean differences, SE: standard error, CI: confidential intervals, SD: standard deviation, I^2^: inconsistency.

**Table 8:** Serum free carnitine (µmol/L) at rest with and without L-carnitine supplementation and serum free carnitine after exercise with and without L-carnitine supplementation.

Authors	Carnitine Dosage		N1	N2	Mean 1	SD 1	Mean 2	SD 2	SMD	SE	95% CI	t	*p*	Weight (%)		Test for Heterogeneity	
														Fixed	Random	Q	72.6140
Arenas 1991 runner	1 g/d, 3 w		13	13	40.0	3	38.0	5	0.470	0.385	−0.326 to 1.265			15.50	9.84	DF	10
Arenas 1991 sprinter	2 g/d, 3 w		11	11	34.0	4	36.0	3	−0.544	0.418	−1.417 to 0.328			13.16	9.70	I^2^ (inconsistency)	86.23%
Arenas 1994	2 g/d, 4 w		7	7	27.8	3.5	31.7	2.9	−1.136	0.544	−2.322 to 0.0504			7.77	9.08	95% CI for I^2^	77.18 to 91.69
Colombani 1996	4 g/d, 1 d		7	7	41.5	2.9	45.8	2.0	−1.616	0.586	−2.893 to −0.339			6.70	8.86		
Cooper 1986	4 g/d, 10 d		10	10	35.4	8.9	33.1	7.9	0.262	0.430	−0.642 to 1.166			12.44	9.64		
Gorostiag 1989	2 g/d, 4 w		10	10	31.4	1.7	39.2	3.3	−2.846	0.621	−4.151 to −1.541			5.97	8.67		
Marconi 1985	4 g/d, 2 w		6	6	48.0	8.3	56.8	9.6	−0.905	0.564	−2.161 to 0.351			7.24	8.98		
Muller 2002	3 g/d, 10 d		10	10	41.10	6.54	52.74	9.23	−1.394	0.482	−2.405 to −0.382			9.92	9.40		
Oyono-Enguelle 1989	2 g/d, 3 w		10	10	49.8	2.3	59.4	3.9	−2.872	0.624	−4.183 to −1.560			5.91	8.66		
Oyono-Enguelle 1989	2 g/d, 4 w		10	10	49.8	2.3	64.1	3.7	−4.445	0.823	−6.175 to −2.716			3.40	7.57		
Oyono-Enguelle 1989	2 g/d, 12 w		10	10	49.8	2.3	48.0	3.5	0.582	0.438	−0.338 to 1.502			12.00	9.61		
Total fixed effects			104	104					−0.787	0.152	−1.086 to −0.488	−5.189	<0.001	100.00	100.00		
Total random effects			104	104					−1.216	0.416	−2.036 to −0.395	−2.921	0.004	100.00	100.00		
**Authors**	**Carn. Dosage**	**Training**	**N1**	**N2**	**Mean 1**	**SD 1**	**Mean 2**	**SD 2**	**SMD**	**SE**	**95% CI**	**t**	***p***	**Weight (%)**		**Test for Heterogeneity**	
														Fixed	Random	Q	24.4106
																DF	6
Arenas 1991 runner	1 g/d, 3 w	After running	13	13	22.9	1.8	25.7	2.6	SMD	SE	95% CI			Weight (%)	SMD	Significance level	*p* = 0.0004
Arenas 1991 sprinter	2 g/d, 3 w	After running	11	11	18.2	1.5	21.0	1.7	−1.680	0.482	−2.686 to −0.675			Fixed	10.38	I^2^ (inconsistency)	75.42%
Colombani1996	4 g/d, 1 d	After running	7	7	45.8	2.0	70.0	4.9	−1.213	0.415	−2.070 to −0.355			20.37	−1.213	95% CI for I^2^	48.00 to 88.38
Cooper 1986	4 g/d, 10 d	After 158′ of Marathon	10	10	22.5	2.8	35.0	12.6	−1.680	0.482	−2.686 to −0.675			15.13	−1.680		
Gorostiaga1989	2 g/d, 4 w	After 40′ of exercise	10	10	35.5	4.4	38.9	2.8	−6.052	1.248	−8.772 to −3.332			2.26	−6.052		
Marconi 1985	4 g/d, 2 w	After 120′ of treadmill	6	6	56.8	9.6	62.5	11.6	−1.312	0.476	−2.311 to −0.312			15.52	−1.312		
Nuesch 1999	1 g/d	10′ after maximal treadmill	9	9	71.3	10.2	71.8	10.7	−0.883	0.450	−1.829 to 0.0635			17.32	−0.883		
Total (fixed effects)			66	66					−0.494	0.542	−1.702 to 0.714	−5.660	<0.001	11.96	−0.494		
Total (random effects)			66	66					−0.0455	0.449	−0.997 to 0.906	−3.253	0.001	17.44	−0.0455		
**Authors**	**Carnitine Dosage**		**N1**	**N2**	**Mean 1**	**SD 1**	**Mean 2**	**SD 2**	**SMD**	**SE**	**95% CI**	**t**	***p***	**Weight (%)**		**Test for Heterogeneity**	
														Fixed	Random		
Arenas 1991 sprinter	2 g/d, 3 w		11	11	34.0	4	36.0	3	−0.544	0.418	−1.417 to 0.328			69.01	51.98	Q	9.5950
Oyono-Enguelle 1989	2 g/d, 3 w		10	10	49.8	2.3	59.4	3.9	−2.872	0.624	−4.183 to −1.560			30.99	48.02	DF	1
Total fixed effects			21	21					−1.266	0.347	−1.968 to −0.563	−3.642	0.001	100.00	100.00	Significance level	*p* = 0.0020
Total random effects			21	21					−1.662	1.163	−4.012 to 0.688	−1.429	0.161	100.00	100.00	I^2^	89.58%
																95% CI for I^2^	61.34 to 97.19
Arenas 1994	2 g/d, 4 w		7	7	27.8	3.5	31.7	2.9	−0.959	0.631	−2.413 to 0.495			41.10	41.10	Q	0.03256
Gorostiaga 1989	2 g/d, 4 w		10	10	31.4	1.7	39.2	3.3	−0.888	0.785	−2.664 to 0.888			26.51	26.51	DF	2
Oyono-Enguelle 1989	2 g/d, 4 w		10	10	49.8	2.3	64.1	3.7	−1.075	0.710	−2.657 to 0.508			32.39	32.39	Significance level	*p* = 0.9839
Total fixed effects			27	27					−0.978	0.404	−1.801 to −0.154	−2.418	0.021	100.00	100.00	I^2^	0.00%
Total random effects			27	27					−0.978	0.404	−1.801 to −0.154	−2.418	0.021	100.00	100.00	95% CI for I^2^	0.00 to 0.00

W: Weeks, g: grams, d: days, l: milliliter, min: minutes, L-carnitine L-carn, SD: standard deviation, N: number of subjects, 1: subjects that did not use carn., 2: subjects used carn, SMD: standard mean differences, SE: standard error, CI: confidential intervals.

## Data Availability

All data generated or analyzed during this study are included in this article.

## References

[1] Chun Y., Lee K., Kang S., Lee N., Kim J. (2011). Influence of L-Carnitine intake for maximal exercise performance and fatigue recovery exercise athletes: Based on elite soccer plaers. Phys. Act. Nutr..

[2] Cruciani R.A., Zhang J.J., Manola J., Cella D., Ansari B., Fisch M.J. (2012). L-Carnitine Supplementation for the Management of Fatigue in Patients with Cancer: An Eastern Cooperative Oncology Group Phase III, Randomized, Double-Blind, Placebo-Controlled Trial. J. Clin. Oncol..

[3] De Simone C., Tzantzoglou S., Famularo G., Moretti S., Paoletti F., Vullo V., Delia S. (1993). High dose L-carnitine improves immunologic and metabolic parameters in AIDS patients. Immunopharmacol. Immunotoxicol..

[4] Siami G., Clinton M.E., Mrak R., Griffis J., Stone W. (1991). Evaluation of the Effect of Intravenous L-Carnitine Therapy on Function, Structure and Fatty Acid Metabolism of Skeletal Muscle in Patients Receiving Chronic Hemodialysis. Nephron.

[5] Silvério R., Laviano A., Fanelli F.R., Seelaender M. (2011). L-carnitine and cancer cachexia: Clinical and experimental aspects. J. Cachex-Sarcopenia Muscle.

[6] Vecchio M., Malaguarnera G., Giordano M., Malaguarnera M., Li Volti G., Galvano F., Drago F., Basile F., Malaguarnera M. (2012). A Musician’s Dystonia. Lancet.

[7] Malaguarnera M., Cammalleri L., Gargante M.P., Vacante M., Colonna V., Motta M. (2007). L-Carnitine treatment reduces severity of physical and mental fatigue and increases cognitive functions in centenarians: A randomized and controlled clinical trial. Am. J. Clin. Nutr..

[8] Kraemer W.J., Volek J.S., Spiering B.A., Vingren J.L. (2005). L-Carnitine Supplementation: A New Paradigm for its Role in Exercise. Mon. Für Chem. /Chem. Mon..

[9] Shamseer L., Moher D., Clarke M., Ghersi D., Liberati A., Petticrew M., Shekelle P., Stewart L.A. (2015). Preferred reporting items for systematic review and meta-analysis protocols (PRISMA-P) 2015: Elaboration and explanation. BMJ.

[10] Stroup D.F., Berlin J.A., Morton S.C., Olkin I., Williamson G.D., Rennie D., Moher D., Becker B.J., Sipe T.A., Thacker S.B. (2000). Meta-analysis of Observational Studies in EpidemiologyA Proposal for Reporting. JAMA.

[11] Methley A.M., Campbell S., Chew-Graham C., McNally R., Cheraghi-Sohi S. (2014). PICO, PICOS and SPIDER: A comparison study of specificity and sensitivity in three search tools for qualitative systematic reviews. BMC Health Serv. Res..

[12] Higgins J.P.T., Green S. (2008). Cochrane Handbook for Systematic Reviews of Interventions.

[13] Guyatt G., Oxman A.D., Akl E.A., Kunz R., Vist G., Brozek J., Norris S., Falck-Ytter Y., Glasziou P., DeBeer H. (2011). GRADE guidelines: 1. Introduction-GRADE evidence profiles and summary of findings tables. J. Clin. Epidemiol..

[14] Guyatt G.H., Oxman A.D., Kunz R., Brozek J., Alonso-Coello P., Rind D., Devereaux P.J., Montori V.M., Freyschuss B., Vist G. (2011). GRADE guidelines 6. Rating the quality of evidence—Imprecision. J. Clin. Epidemiol..

[15] Guyatt G.H., Oxman A.D., Vist G., Kunz R., Brozek J., Alonso-Coello P., Montori V., Akl E.A., Djulbegovic B., Falck-Ytter Y. (2011). GRADE guidelines: 4. Rating the quality of evidence—Study limitations (risk of bias). J. Clin. Epidemiol..

[16] Guyatt G.H., Oxman A.D., Montori V., Vist G., Kunz R., Brozek J., Alonso-Coello P., Djulbegovic B., Atkins D., Falck-Ytter Y. (2011). GRADE guidelines: 5. Rating the quality of evidence—Publication bias. J. Clin. Epidemiol..

[17] Guyatt G.H., Oxman A.D., Kunz R., Woodcock J., Brozek J., Helfand M., Alonso-Coello P., Falck-Ytter Y., Jaeschke R., Vist G. (2011). GRADE guidelines: 8. Rating the quality of evidence—indirectness. J. Clin. Epidemiol..

[18] Huedo-Medina T.B., Sánchez-Meca J., Marín-Martínez F., Botella J. (2006). Assessing heterogeneity in meta-analysis: Q statistic or I^2^ index?. Psychol. Methods.

[19] DerSimonian R., Kacker R. (2007). Random-effects model for meta-analysis of clinical trials: An update. Contemp. Clin. Trials.

[20] Higgins J.P., Altman D.G., Gøtzsche P.C., Jüni P., Moher D. (2011). The Cochrane Collaboration’s tool for assessing risk of bias in randomised trials. Br. Med. J..

[21] Brass E.P., Hoppel C.L., Hiatt W.R. (1994). Effect of intravenous L-carnitine on carnitine homeostasis and fuel metabolism during exercise in humans. Clin. Pharmacol. Ther..

[22] Colombani P., Wenk C., Kunz I., Krähenbühl S., Kuhnt M., Arnold M., Frey-Rindova P., Frey W., Langhans W. (1996). Effects of L-carnitine supplementation on physical performance and energy metabolism of endurance-trained athletes: A double-blind crossover field study. Eur. J. Appl. Physiol. Occup. Physiol..

[23] Marconi C., Sassi G., Carpinelli A., Cerretelli P. (1985). Effects of L-carnitine loading on the aerobic and anaerobic performance of endurance athletes. Eur. J. Appl. Physiol. Occup. Physiol..

[24] Greig C., Finch K.M., Jones D.A., Cooper M., Sargeant A.J., Forte C.A. (1987). The effect of oral supplementation with l-carnitine on maximum and submaximum exercise capacity. Graefe’s Arch. Clin. Exp. Ophthalmol..

[25] Koozehchian M.S., Daneshfar A., Fallah E., Agha-Alinejad H., Samadi M., Kaviani M., Kaveh B M., Jung Y.P., Sablouei M.H., Moradi N. (2018). Effects of nine weeks L-Carnitine supplementation on exercise performance, anaerobic power, and exercise-induced oxidative stress in resistance-trained males. J. Exerc. Nutr. Biochem..

[26] Vecchiet L., Di Lisa F., Pieralisi G., Ripari P., Menabò R., Giamberardino M.A., Siliprandi N. (1990). Influence of L-carnitine administration on maximal physical exercise. Eur. J. Appl. Physiol. Occup. Physiol..

[27] Stuessi C., Hofer P., Meier C., Boutellier U. (2005). L -Carnitine and the recovery from exhaustive endurance exercise: A randomised, double-blind, placebo-controlled trial. Eur. J. Appl. Physiol..

[28] Orer G.E., Guzel N.A. (2014). The Effects of Acute L-carnitine Supplementation on Endurance Performance of Athletes. J. Strength Cond. Res..

[29] Siliprandi N., Di Lisa F., Pieralisi G., Ripari P., Maccari F., Menabo R., Giamberardino M.A., Vecchiat L. (1990). Metabolic changes induced by maximal exercise in human subjects following L-carnitine administration. Biochim. Biophys. Acta (BBA)-Gen. Subj..

[30] Soop M., Björkman O., Cederblad G., Hagenfeldt L., Wahren J. (1988). Influence of carnitine supplementation on muscle substrate and carnitine metabolism during exercise. J. Appl. Physiol..

[31] Wyss V., Ganzit G.P., Rienzi A. (1990). Effects of L-carnitine administration on VO2max and the aerobic-anaerobic threshold in normoxia and acute hypoxia. Eur. J. Appl. Physiol. Occup. Physiol..

[32] Mor A., Baynaz K., Ipekoglu G., Arslanoglu C., Acar K., Cakir H.I., Arslanoglu E. (2018). Effect of L-Carnitine Supplementation on Weight Loss and Body Composition of Taekwondo Players. J. Sports Educ..

[33] Cooper M.B., Jones D.A., Edwards R.H.T., Corbucci C., Montanari G., Trevisani C. (1986). The effect of marathon running on carnitine metabolism and on some aspects of muscle mitochondrial activities and antioxidant mechanisms. J. Sports Sci..

[34] Müller D.M., Seim H., Kiess W., Ster H.L., Richter T. (2002). Effects of oral L-carnitine supplementation on in vivo long-chain fatty acid oxidation in healthy adults. Metabolism.

[35] Parandak K., Arazi H., Khoshkhahesh F., Nakhostin-Roohi B. (2014). The Effect of Two-Week L-Carnitine Supplementation on Exercise -Induced Oxidative Stress and Muscle Damage. Asian J. Sports Med..

[36] Giamberardino M.A., Dragani L., Valente R., Di Lisa F., Saggin R., Vecchiet L. (1996). Effects of Prolonged L-Carnitine Administration on Delayed Muscle Pain and CK Release After Eccentric Effort. Endoscopy.

[37] Arenas J., Huertas R., Campos Y., Díaz A.E., Villalón J.M., Vilas E. (1994). Effects of L-carnitine on the pyruvate dehydrogenase complex and carnitine palmitoyl transferase activities in muscle of endurance athletes. FEBS Lett..

[38] Gorostiaga E., Maurer C., Eclache J. (1989). Decrease in Respiratory Quotient During Exercise Following L-Carnitine Supplementation. Int. J. Sports Med..

[39] Huertas R., Campos Y., Díaz E., Esteban J., Vechietti L., Montanari G., D’Iddio S., Corsi M., Arenas J. (1992). Respiratory chain enzymes in muscle of endurance athletes: Effect of L-carnitine. Biochem. Biophys. Res. Commun..

[40] Oyono-Enguelle S., Freund H., Ott C., Gartner M., Heitz A., Marbach J., Maccari F., Frey A., Bigot H., Bach A.C. (1988). Prolonged submaximal exercise and L-carnitine in humans. Graefe’s Arch. Clin. Exp. Ophthalmol..

[41] Swart I., Rossouw J., Loots J., Kruger M. (1997). The effect of L-carnitine supplementation on plasma carnitine levels and various performance parameters of male marathon athletes. Nutr. Res..

[42] Badrasawi M., Shahar S., Zahara A.M., Nor Fadilah R., Singh D.K. (2016). Efficacy of L-carnitine supplementation on frailty status and its biomarkers, nutritional status, and physical and cognitive function among prefrail older adults: A double-blind, randomized, placebo-controlled clinical trial. Clin. Interv. Aging.

[43] Wächter S., Vogt M., Kreis R., Boesch C., Bigler P., Hoppeler H., Krähenbühl S. (2002). Long-term administration of l-carnitine to humans: Effect on skeletal muscle carnitine content and physical performance. Clin. Chim. Acta.

[44] DrĂgan I.G., Vasiliu A., Georgescu E., Eremia N. (1989). Studies concerning chronic and acute effects of L-carnitina in elite athletes. Physiologie.

[45] Natali A., Santoro D., Brandi L.S., Faraggiana D., Ciociaro D., Pecori N., Buzzigoli G., Ferrannini E. (1993). Effects of acute hypercarnitinemia during increased fatty substrate oxidation in man. Metabolism.

[46] Pistone G., Marino A., Leotta C., Dell’Arte S., Finocchiaro G., Malaguarnera M. (2003). Levocarnitine administration in elderly subjects with rapid muscle fatigue: Effect on body composition, lipid profile and fatigue. Drugs Aging.

[47] Nüesch R., Rossetto M., Martina B. (1999). Plasma and urine carnitine concentrations in well-trained athletes at rest and after exercise. Influence of L-carnitine intake. Drugs Exp. Clin. Res..

[48] Hiatt W.R., Regensteiner J.G., Wolfel E.E., Ruff L., Brass E.P. (1989). Carnitine and acylcarnitine metabolism during exercise in humans. Dependence on skeletal muscle metabolic state. J. Clin. Investig..

[49] Harris R.C., Foster C.V., Hultman E. (1987). Acetylcarnitine formation during intense muscular contraction in humans. J. Appl. Physiol..

[50] Carlin J.I., Reddan W.G., Sanjak M., Hodach R. (1986). Carnitine metabolism during prolonged exercise and recovery in humans. J. Appl. Physiol..

[51] Janssen G.M.E., Scholte H.R., Vaandrager-Verduin M.H.M., Ross J.D. (1989). Muscle Carnitine Level in Endurance Training and Running a Marathon. Int. J. Sports Med..

[52] Arenas J., Ricoy J.R., Encinas A.R., Pola P., D’Iddio S. (1991). Carnitine in muscle, serum, and urine of nonprofessional athletes: Effects of physical exercise, training, and L-carnitine administration. Muscle Nerve.

[53] Drăgan G.I., Vasiliu A., Georgescu E., Dumas I. (1987). Studies concerning chronic and acute effects of L-carnitine on some biological parameters in elite athletes. Physiologie.

[54] Drăgan G.I., Wagner W., Ploeşteanu E. (1988). Studies concerning the ergogenic value of protein supply and 1-carnitine in elite junior cyclists. Physiologie.

[55] Geidl W., Wais J., Fangmann C., Demisse E., Pfeifer K., Sudeck G. (2019). Physical activity promotion in daily exercise therapy: The perspectives of exercise therapists in German rehabilitation settings. BMC Sports Sci. Med. Rehabil..

[56] Constantin-Teodosiu D., Carlin J.I., Cederblad G., Harrist R.C., Hultman E. (1991). Acetyl group accumulation and pyruvate dehydrogenase activity in human muscle during incremental exercise. Acta Physiol. Scand..

[57] Lennon D.L., Stratman F.W., Shrago E., Nagle F.J., Madden M., Hanson P., Carter A.L. (1983). Effects of acute moderate-intensity exercise on carnitine metabolism in men and women. J. Appl. Physiol..

[58] Malaguarnera G., Catania V.E., Bonfiglio C., Bertino G., Vicari E., Malaguarnera M. (2020). Carnitine Serum Levels in Frail Older Subjects. Nutrients.

[59] Roepstorff C., Halberg N., Hillig T., Saha A.K., Ruderman N.B., Wojtaszewski J.F.P., Richter E.A., Kiens B. (2005). Malonyl-CoA and carnitine in regulation of fat oxidation in human skeletal muscle during exercise. Am. J. Physiol. Metab..

[60] Maughan R.J. (1999). Nutritional ergogenic aids and exercise performance. Nutr. Res. Rev..

